# The lupus autoantigen La/Ssb is an *Xist*-binding protein involved in *Xist* folding and cloud formation

**DOI:** 10.1093/nar/gkab1003

**Published:** 2021-11-01

**Authors:** Norbert Ha, Nan Ding, Ru Hong, Rubing Liu, Xavier Roca, Yingyuan Luo, Xiaowei Duan, Xiao Wang, Peiling Ni, Haiyang Wu, Li-Feng Zhang, Lingyi Chen

**Affiliations:** School of Biological Sciences, Nanyang Technological University, 60 Nanyang Drive, Singapore 637551; Institute of Translational Medicine, Tianjin Union Medical Center, State Key Laboratory of Medicinal Chemical Biology, Collaborative Innovation Center for Biotherapy, Collaborative Innovation Center of Tianjin for Medical Epigenetics, Tianjin Key Laboratory of Protein Sciences, National Demonstration Center for Experimental Biology Education and College of Life Sciences, Nankai University, Tianjin 300071, China; School of Biological Sciences, Nanyang Technological University, 60 Nanyang Drive, Singapore 637551; School of Biological Sciences, Nanyang Technological University, 60 Nanyang Drive, Singapore 637551; School of Biological Sciences, Nanyang Technological University, 60 Nanyang Drive, Singapore 637551; Institute of Translational Medicine, Tianjin Union Medical Center, State Key Laboratory of Medicinal Chemical Biology, Collaborative Innovation Center for Biotherapy, Collaborative Innovation Center of Tianjin for Medical Epigenetics, Tianjin Key Laboratory of Protein Sciences, National Demonstration Center for Experimental Biology Education and College of Life Sciences, Nankai University, Tianjin 300071, China; Institute of Translational Medicine, Tianjin Union Medical Center, State Key Laboratory of Medicinal Chemical Biology, Collaborative Innovation Center for Biotherapy, Collaborative Innovation Center of Tianjin for Medical Epigenetics, Tianjin Key Laboratory of Protein Sciences, National Demonstration Center for Experimental Biology Education and College of Life Sciences, Nankai University, Tianjin 300071, China; Institute of Translational Medicine, Tianjin Union Medical Center, State Key Laboratory of Medicinal Chemical Biology, Collaborative Innovation Center for Biotherapy, Collaborative Innovation Center of Tianjin for Medical Epigenetics, Tianjin Key Laboratory of Protein Sciences, National Demonstration Center for Experimental Biology Education and College of Life Sciences, Nankai University, Tianjin 300071, China; Institute of Translational Medicine, Tianjin Union Medical Center, State Key Laboratory of Medicinal Chemical Biology, Collaborative Innovation Center for Biotherapy, Collaborative Innovation Center of Tianjin for Medical Epigenetics, Tianjin Key Laboratory of Protein Sciences, National Demonstration Center for Experimental Biology Education and College of Life Sciences, Nankai University, Tianjin 300071, China; TCRCure Biological Technology Co Ltd., Guangdong, China; School of Biological Sciences, Nanyang Technological University, 60 Nanyang Drive, Singapore 637551; TCRCure Biological Technology Co Ltd., Guangdong, China; Institute of Translational Medicine, Tianjin Union Medical Center, State Key Laboratory of Medicinal Chemical Biology, Collaborative Innovation Center for Biotherapy, Collaborative Innovation Center of Tianjin for Medical Epigenetics, Tianjin Key Laboratory of Protein Sciences, National Demonstration Center for Experimental Biology Education and College of Life Sciences, Nankai University, Tianjin 300071, China

## Abstract

Using the programmable RNA-sequence binding domain of the Pumilio protein, we FLAG-tagged *Xist* (inactivated X chromosome specific transcript) in live mouse cells. Affinity pulldown coupled to mass spectrometry was employed to identify a list of 138 candidate *Xist*-binding proteins, from which, Ssb (also known as the lupus autoantigen La) was validated as a protein functionally critical for X chromosome inactivation (XCI). Extensive XCI defects were detected in *Ssb* knockdown cells, including chromatin compaction, death of female mouse embryonic stem cells during *in vitro* differentiation and chromosome-wide monoallelic gene expression pattern. Live-cell imaging of *Xist* RNA reveals the defining XCI defect: *Xist* cloud formation. Ssb is a ubiquitous and versatile RNA-binding protein with RNA chaperone and RNA helicase activities. Functional dissection of Ssb shows that the RNA chaperone domain plays critical roles in XCI. In Ssb knockdown cells, *Xist* transcripts are unstable and misfolded. These results show that Ssb is critically involved in XCI, possibly as a protein regulating the in-cell structure of *Xist*.

## INTRODUCTION

*Xist* RNA is a prototype long non-coding RNA (lncRNA) involved in X chromosome inactivation (XCI), a mammalian dosage compensation mechanism, in which one female X chromosome is transcriptionally silenced to balance the X-linked gene dosage between males and females (1). Upon the onset of XCI during early embryonic development, the coating of *Xist* RNA transcripts on the chromosome territory of the chosen inactive X chromosome (Xi) recruits epigenetic factors for heterochromatinization and establishes the chromosome-wide gene silencing (1). Intensive efforts have been spent in isolating *Xist*-binding proteins. Three attempts of comprehensive isolation of *Xist*-binding proteins were reported in 2015 (2–4). Chu *et al.* identified 81 candidate *Xist*-binding proteins (4). Using a more selective approach, McHugh et al identified a list of 10 candidate proteins (3). Meanwhile, through a more sensitive approach, Minajigi et al. generated a list of >700 proteins (2). 4 proteins were commonly identified by all three studies. These results generate an initial portrait of a fascinating and yet still poorly understood epigenetic machinery recruited/assembled by the *Xist* RNA. These results also illustrate the difficulty of balancing the assay's specificity and sensitivity in profiling a complex lncRNA–protein interactome.

Advanced by breakthrough technologies in isolating lncRNA-binding proteins, the list of key proteins recruited by *Xist* is growing, which includes transcription repressors (SPEN, also known as SHARP, SMRT/HDAC1-associated repressor protein), proteins involved in N^6^-adenosine (m^6^A) RNA methylation (RBM15 and WTAP), heterogeneous nuclear ribonucleoproteins (hnRNP K and hnRNP U), histone modifiers (polycomb proteins) (2–9). It is believed that *Xist* recruits the proteins and functions as a scaffold to assemble them into a protein machinery. However, the portrait of this protein machinery remains fragmented. Many questions remain elusive.

**Figure 1.**
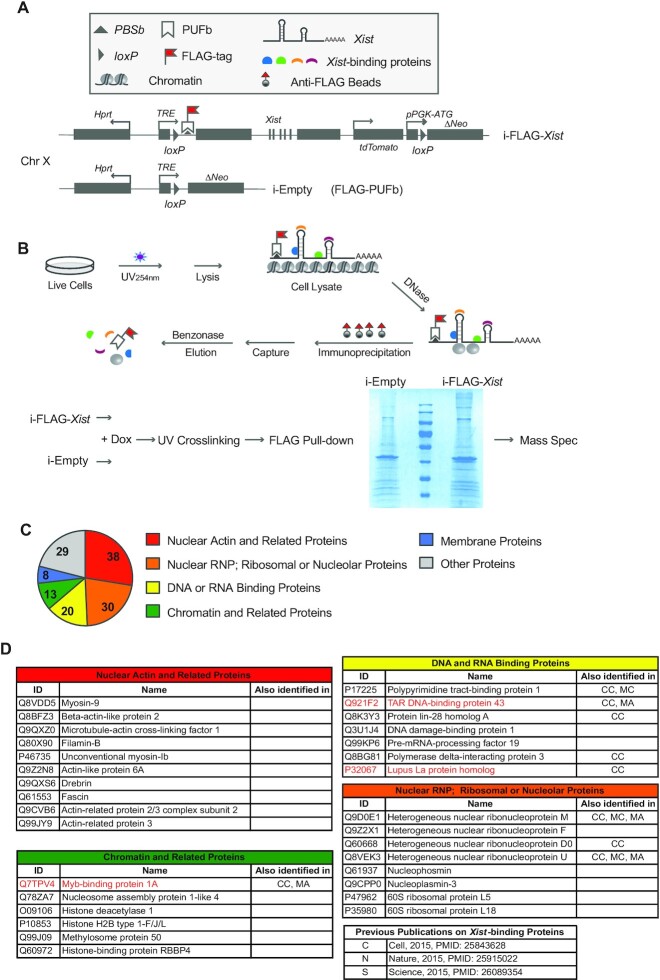
Schematic diagram of ‘FLAG-out’ and its identified *Xist*-binding proteins. (**A**) Diagram of the transgenic mouse ES cell lines i-FLAG-*Xist* and i-Empty. Both cell lines stably express PUFb-FLAG fusion protein, which binds RNA at the PBSb site. In addition, the i-FLAG-*Xist* ES cell line carries an X-linked single-copy inducible *Xist* transgene with 25 tandem PBSb sites fused to its 5′-ends. (**B**) Diagram of the experimental design of ‘FLAG-out’ and a protein gel image of the FLAG pull-down samples. Taking advantage of the binding between PUFb and PBSb, immunoprecipitation with FLAG antibody allows pulling down PUFb-FLAG and PBSb-*Xist* RNA, as well as *Xist* binding proteins. (**C**, **D**) Candidate *Xist*-binding proteins identified by FLAG-out. CC: Chu C *et al.* (4), MC: McHugh CA *et al.* (3), MA: Minajigi A *et al.* (2).

Here, we devised a different system for profiling the *Xist* interactome. We have previously shown that the *Xist* RNA can be efficiently tagged in live mouse cells by the programmable RNA-sequence binding domain of the Pumilio protein (PUF: pumilio-homology domain; PBS: PUF-binding site) (10). A transgenic cell line previously generated in the lab is a male mouse embryonic stem (ES) cell line carrying an X-linked single-copy inducible *Xist* transgene with 25 tandem PBSb sites fused to its 5′-ends (Figure 1A) (10). We further engineered the cell line to have it stably express a PUFb-FLAG fusion protein, which recognizes the PBSb site (i-FLAG-*Xist*, Figure 1A). Therefore, in this cell line, the *Xist* RNA is efficiently FLAG-tagged, which enables the usage of the highly specific and sensitive anti-FLAG antibody in the subsequent protein isolation work (Figure 1B). Meanwhile, we engineered a negative control cell line, i-Empty, which stably expresses PUFb-FLAG but carries an empty inducible cassette (Figure 1A). We name this method ‘FLAG-out’. In this study, using FLAG-out, we identified 138 candidate *Xist*-binding proteins, and further validated Ssb (also known as the lupus autoantigen La) as an *Xist*-binding protein functionally critical for XCI.

## MATERIAL AND METHODS

### Cell lines and culture

Mouse ES cells were cultured in medium containing 1000 unit/ml LIF. Feeder cells used were Drug Resistant 4 Mouse Embryonic Fibroblasts (DR4-MEF) (Applied StemCell; ASF-1002). Feeder-free ES cells were cultured on culture dishes pre-treated with 0.2% gelatin (Sigma-Aldrich; G2500).

For *in vitro* differentiation, cells were cultured in differentiation medium containing 50 μg/ml L-ascorbic acid (Sigma). For the first 4 days of *in vitro* differentiation, embryoid bodies (EBs) were cultured in suspension. On day 4, EBs were transferred to a gelatinized T75 tissue culture flask. On day 5, EBs failed to attach to the tissue culture flask were washed away. On day 6, surviving cells were harvested for subsequent experiments.

### FLAG-out

Three 15 cm dishes of ES cells were cultured in differentiation medium supplemented with doxycycline for 48 h. Cells were washed with ice cold PBS once and crosslinked with 0.4 J/cm^2^ of UV_254 nm_. Cells were then harvested by trypsin treatment and lysed in 3 ml of lysis buffer (50 mM Tris–HCl, pH 7.4; 150 mM NaCl; 1% TRITON X-100; 5% glycerol; supplemented with protease inhibitors and RNase Inhibitor). Cell lysates were first digested with TURBO DNase (Ambion). After centrifugation, supernatants were collected for co-IP experiment using anti-Flag M2 beads (Sigma). Elution of IPed proteins from anti-Flag M2 beads was achieved by five rounds of RNA/DNA digestion using 250 U of benzonase (Millipore, #71206-3) for 2 h at 37°C. The eluted proteins were concentrated in a speed-vacuum to a final volume about 60 μl. One third of the elusions were separated on a 10% SDS-PAGE gel and stained by colloidal blue. The rest of the elusions were run on another 10% SDS-PAGE gel shortly, and the unresolved gel slices were subjected to in-gel trypsin digestion and liquid chromatograph-tandem mass spectrometry (MS/MS) using Orbitrap FusionTM (Thermo) (Performed by PTM Bio, Hangzhou, China). For proteins identified in both i-FLAG-*Xist* and i-Empty samples, ranking numbers were assigned according to the ranks of their protein scores (The sum of the ion scores of all peptides that were identified) in each sample (Supplementary Table S2D). The ranking gains were calculated as: the rank number in i-Empty - the rank number in i-FLAG-*Xist*, similar to the method described in the previous publication (4).

### Cell Survival Assay

Doxycycline treatment of 1 μg/ml was used throughout the study. G418 (ThermoFisher) treatment was carried out at 250 μg/ml. If ES cells were cultured as undifferentiated ES cells, Alkaline phosphatase (AP) staining (Vector Laboratories) was used at the end of the assay for counting the cells. After AP staining, milk is added to the well to provide a white background for picture taking, then the whole tissue culture plate is put onto a scanner to scan the image. ImageJ (11) was used to analyze AP staining data.

### shRNA knockdown

An shRNA system (OligoEngine, pSUPER RNAi System) was used. The following shRNA sequences were designed against *Mybbp1a* (5′-CCGGAGTGTATTTGGTCATAT-3′), *Tardbp* (5′-GAATATGAAACCCAAGTGAAA-3′), *Ssb*-1(5′-GAGAAGATTTACACTTCCTTT-3′), and *Ssb-*2 (5′-GAACAGATCAAATTGGATGAA-3′). 300 μg/ml Hygromycin B (ThermoFisher; 10687010) was used to select stably transfected cell lines. Single surviving ES cell colonies were picked and the knockdown efficiency was validated using RT-qPCR.

### Quantitative RT-PCR

Total RNA was isolated by TRIzol (Life technologies). cDNA was synthesized using iScript reverse transcription kit (170–8840, Bio-Rad). The real-time PCR was carried out on the CFX Connect real-time PCR system (Bio-Rad) using the SsoAdvanced Universal SYBR Green Supermix (Bio-Rad). The following PCR primers were used: Actb (F: 5′- ACTGCCGCATCCTCTTCCTC-3′, R: 5′-CCGCTCGTTGCCAATAGTGA-3′); Gapdh (F: 5′-CCAATGTGTCCGTCGTGGAT-3′, R: 5′-TGCCTGCTTCACCACCTTCT-3′); Mybbp1a (F: 5′-GCCGCGAGTTCTTGGACTT-3′, R:5′-ATCTCCGAATCATTGGGCCTT-3′); Tardbp (F: 5′- AATCAGGGTGGGTTTGGTAACA-3′, R: 5′-GCTGGGTTAATGCTAAAAGCAC-3′); Ssb (F: 5′-ACCAAGCAGACCACTCCCTG-3′, R: 5′-GGTGGCGTCAGTTGGGAAAC-3′); Xist E1 (F: 5′-CGGCCTCTAGTTTGTCCATT-3′, R: 5′-GATGGCATGATGGAATTGAG-3′); Xist E7 (F: 5′-TCCTACCATCATCTGCTTTGA-3′, R: 5′-CATCCGTTCACAAAGTCCAG-3′); GFP (F: 5′-GATGAAGAGCACCAAAGGCGC-3′, R: 5′-CCGTTGTTGATGGCGTGCAG-3′).

### Padlock probe SNP capture

The padlock probe library design and padlock SNP capture procedure were performed as previously described (12). Briefly, each reaction was carried out in 20 μl volume containing 1 unit Ampligase (A3210K, Epicenter), 1 unit Phusion High-Fidelity DNA Polymerase (M0530, New England BioLabs), 1× Phusion High-Fidelity DNA Polymerase buffer, 10 nM dNTP. cDNA was generated using SuperScript III first-strand synthesis system (18080-051, Life Technologies). Two hundred nanograms of single-stranded cDNA and 2 pmol padlock probe were used in each reaction. Nicotinamide adenine dinucleotide (Sigma; N0632) was provided in each reaction at a final concentration of 0.5 mM.

The multiplexed sequencing libraries were PCR amplified in a real-time PCR system (CFX Connect, Bio-Rad) using the following primers: CA-2-RA.Miseq (5′-AATGATACGGCGACCACCGAGATCTATCGGCTACACGCCTATCGGGAAGCTGAAG-3′); CA-2-FA.Indx7Sol (5′-CAAGCAGAAGACGGCATACGAGATGATCTGCGGTCTGCCATCCGACGGTAGTGT-3′); CA-2-FA.Indx45Sol (5′-CAAGCAGAAGACGGCATACGAGATCGTAGTCGGTCTGCCATCCGACGGTAGTGT-3′); CA-2-FA.Indx76Sol (5′-CAAGCAGAAGACGGCATACGAGATAATAGGCGGTCTGCCATCCGACGGTAGTGT-3′); CA-2-FA.Indx91Sol (5′-CAAGCAGAAGACGGCATACGAGATACATCGCGGTCTGCCATCCGACGGTAGTGT-3′); CA-2-FA.Indx92Sol (5′-CAAGCAGAAGACGGCATACGAGATTCAAGTCGGTCTGCCATCCGACGGTAGTGT-3′); CA-2-FA.Indx93Sol (5′-CAAGCAGAAGACGGCATACGAGATATTGGCCGGTCTGCCATCCGACGGTAGTGT-3′). The following sequencing primers were used: Read1.Miseq (5′-ATCGGCTACACGCCTATCGGGAAGCTGAAG-3′); IndexRead (5′-ACACTACCGTCGGATGGCAGACCG-3′). Sequencing was carried out on an Illumina NextSeq system.

### ATAC-seq

Trypsinized cells were pelleted and washed with PBS. A total of 55,000 cells were lysed using cold lysis buffer (10 mM Tris–HCl, pH 7.4, 10 mM NaCl, 3 mM MgCl_2_, 0.1% (v/v) Igepal CA-630) for 3 mins and centrifuged at 500 × g at 4°C for 10 min. Pellets were resuspended with transposition reaction mix (2.5 μl TD, 2.5 μl TDE1, 22.5 μl nuclease-free water) (Nextera DNA library preparation kit, Cat. No. FC-121-1030) and incubated in Thermomixer at 37°C for 30 min with 1000 rpm. Transposed DNA samples were purified with Qiagen MinElute PCR purification kit (Cat. No.: 28006) and eluted with 20 μl elution buffer from Qiagen MinElute PCR purification kit. The purified transposed DNA fragments were amplified by PCR. Each reaction was carried out in 50 μl volume containing 20 μl of transposed DNA, 2.5 μl of 25 μM Ad1_noMX primer (13), 2.5 μl of 25 μM Ad2.* indexing primer (13), 25 μl of NEBNext High-Fidelity 2× PCR Master Mix (Cat. No. M0541) with the following cycles: 1 cycle: 5 min at 72 °C, 30 s at 98 °C and 5 cycles: 10 s at 98 °C, 30 s at 63 °C, 1 min at 72 °C. Quantitative PCR was carried out on the PCR products to calculate the additional PCR cycles required before reaching saturation. Each qPCR reaction was carried out in 15 μl volume containing 5 μl of partially PCR-amplified DNA, 4.41 μl of nuclease-free H_2_O, 0.25 μl of 25 μM Ad1_noMX primer, 0.25 μl of 25 μM Ad2.* indexing primer, 0.09 μl of 100× SYBR Green I (Cat. No. S-7563), 5 μl of NEBNext High-Fidelity 2× PCR Master Mix (Cat. No. M0541) with the following cycles: 1 cycle: 30 s at 98 °C and 20 cycles: 10 s at 98 °C, 30 s at 63 °C, 1 min at 72 °C. The additional number of PCR cycles (*N*) is the cycle that corresponds to one-third of the maximum fluorescent intensity. After determining the additional PCR cycles required, the remaining 45 μl of transposed DNA was further amplified with the following cycles: 1 cycle: 30 s at 98 °C and N cycles: 10 s at 98 °C, 30 s at 63 °C, 1 min at 72 °C. ATAC-seq libraries were two-stage purified using AMPure XP beads: 0.5× and 1.3×. Libraries are eluted in 20 μl nuclease-free water. The size and pattern of the ATAC-seq libraries were assessed by 6% TBE-PAGE. The concentration of each library sample was measured by KAPA Library Quantification kit (Cat. No.: kk4824). All libraries were pooled and a final quality check was performed using Bioanalyzer High-Sensitivity DNA Analysis kit. Sequencing was carried out on Illumina HiSeq 4000 system.

The followings were the primers used in this study:

Ad1_noMX (5′-AATGATACGGCGACCACCGAGATCTACACTCGTCGGCAGCGTCAGATGTG-3′); Ad2.1_TAAGGCGA(5′-CAAGCAGAAGACGGCATACGAGATTCGCCTTAGTCTCGTGGGCTCGGAGATGT-3′); Ad2.2_CGTACTAG (5′-CAAGCAGAAGACGGCATACGAGATCTAGTACGGTCTCGTGGGCTCGGAGATGT-3′); Ad2.3_AGGCAGAA (5′-CAAGCAGAAGACGGCATACGAGATTTCTGCCTGTCTCGTGGGCTCGGAGATGT-3′) Ad2.4_TCCTGAGC (5′-CAAGCAGAAGACGGCATACGAGATGCTCAGGAGTCTCGTGGGCTCGGAGATGT-3′) Ad2.5_GGACTCCT (5′-CAAGCAGAAGACGGCATACGAGATAGGAGTCCGTCTCGTGGGCTCGGAGATGT-3′) Ad2.6_TAGGCATG (5′-CAAGCAGAAGACGGCATACGAGATCATGCCTAGTCTCGTGGGCTCGGAGATGT-3′); Ad2.7_CTCTCTAC (5′-CAAGCAGAAGACGGCATACGAGATGTAGAGAGGTCTCGTGGGCTCGGAGATGT-3′); Ad2.8_CAGAGAGG (5′-CAAGCAGAAGACGGCATACGAGATCCTCTCTGGTCTCGTGGGCTCGGAGATGT-3′); Ad2.9_GCTACGCT (5′-CAAGCAGAAGACGGCATACGAGATAGCGTAGCGTCTCGTGGGCTCGGAGATGT-3′) Ad2.10_CGAGGCTG (5′-CAAGCAGAAGACGGCATACGAGATCAGCCTCGGTCTCGTGGGCTCGGAGATGT-3′).

### RNA immunoprecipitation (RIP)

Empty pCAG-IPuro vector or pCAG-IPuro expressing FLAG-Ssb was transfected into *Xist* inducible male ES cells. And stable cell lines were established after puromycin selection. Three 15-cm dishes of ES cells were harvested, crosslinked with 1% formaldehyde for 10 min at room temperature, and quenched with 0.125 M glycine for 5 min. After centrifugation, cell pellets were resuspended in lysis buffer (50 mM Tris–HCl pH 7.4, 150 mM NaCl, 1% TRITON X-100, 5% glycerol, 1 mM PMSF, 1 mM DTT, 1.52 mg/ml protease inhibitor cocktail, 400 U/ml RNase inhibitor). Incubate the cells for 30–60 min on a rotor at 4°C. Treat the lysate with RQ1 DNase (Promega M6101) at 37°C for 10 min. Insoluble material was removed by centrifugation, and the supernatant was incubated with anti-FLAG M2 beads (Sigma M8823) overnight at 4°C. The beads were washed with IP 200 buffer (20 mM Tris–HCl pH 7.4, 200 mM NaCl, 1 mM EDTA, 0.3% TRITON X-100, 5% Glycerol) for five times. RNA-protein complexes were eluted from FLAG-beads by adding Proteinase K digestion buffer (50 mM Tris–HCl pH 7.4, 10 mM EDTA, 50 mM NaCl, 0.5% SDS, 400 μg/ml Proteinase K (Roche 3115844001)), and incubated at 55°C for 1 h. Then RNA was extracted using Trizol (Invitrogen). Quantitative RT-PCR was performed to quantify the amount of *Xist, 7SK* and *Actin* RNA. The following PCR primers were used: *Xist*-1 (F: 5′- GGACATTTAGGTCGTACAGGAA-3′, R: 5′-CCAGGCTAACTCAGACATGAAG-3′); *Xist*-2 (F: 5′-TGCATACGGTCCTATTGCTAAA-3′, R: 5′- CCATGACTCTGGAAGTCAGTATG-3′); *Xist*-3 (F: 5′-CCTGCAAGGGATACCGTTTAT-3′, R: 5′-ATGAAAGGCGAAGGAGTATGG-3′); *Xist*-4 (F: 5′- AGAGTTGTTCCAGACCAATGA-3′, R: 5′-CTCACCTAAGCCCAAAGAAAGA-3′); *Xist*-5 (F: 5′-CCATGTGGTTGTTGGGATTTG-3′, R: 5′- TTACTCAGAAGGCTGGAGAGA-3′); *Actb* (F: 5′-ACTGCCGCATCCTCTTCCTC-3′, R: 5′-CCGCTCGTTGCCAATAGTGA-3′); *7SK* (F: 5′-TCGGTCAAGGGTATACGAGTAG-3′, R: 5′-TTTGGATGTGTCTGGAGTCTTG-3′).

### CLIP

Three 15 cm dishes of WT ES cells or ES cells stably expressing FLAG-Ssb were treated with Dox for 24 h. Medium was removed and replaced by ice-cold PBS. Then the cells were irradiated with 0.4 J/cm^2^ of UV_254 nm_, collected and resuspended in 1 ml lysis buffer (50 mM Tris–HCl pH 7.4, 100 mM NaCl, 1% NP-40, 0.1% SDS, 0.5% sodium deoxycholate, 1.52 mg/ml protease inhibitor cocktail). After a 15-min incubation on ice, the lysate was treated with 2 μl RQ1 DNase and 10 μl RNase I (0.001 U/μl), and incubated for 5 min at 37°C on the mixer. Insoluble material was removed by centrifugation, and the supernatant was incubated with anti-FLAG M2 beads overnight at 4°C. The beads were washed with 1 ml high salt wash buffer (50 mM Tris–HCl pH 7.4, 1 M NaCl, 1% NP-40, 0.1% SDS, 0.5% sodium deoxycholate, 1 mM EDTA) for two times; 500 μl wash buffer (20 mM Tris–HCl pH 7.4, 10 mM MgCl_2_, 0.2% Tween-20) for one time; 1 ml IP 200 buffer (20 mM Tris–HCl pH 7.4, 200 mM NaCl, 0.3% Triton X-100, 5% glycerol, 1 mM EDTA) for three times. RNA-protein complexes were eluted from FLAG-beads by adding 200 μl Proteinase K digestion buffer (100 mM Tris–HCl pH 7.4, 10 mM EDTA, 50 mM NaCl, 10 μl Proteinase per 200 μl buffer), and incubated 20 min at 37°C. Next, 200 μl PK urea buffer (100 mM Tris–HCl pH 7.4, 10 mM EDTA, 50 mM NaCl, 7 M urea) was added and incubated 20 min at 37°C. Then RNA was extracted using Trizol. Quantitative RT-PCR was performed to quantify the amount of Xist, 7SK, and Actin RNA. Primers were described in the RIP section. Additional PCR primers were used: *Xist*-1a (F: 5′-GCTTGGTGGATGGAAATATGG-3′, R: 5′-CCGCAAATGCTACCACAAATC-3′); *Xist*-1b (F: 5′-TGCCCTCTAGTGGTTTCTTTC-3′, R: 5′-CTAAAGACACACGTGAAGTACCAAG-3′).

### SHAPE-MaP

SHAPE-MaP was performed as described elsewhere (14,15). Briefly, ES cells were washed once with phosphate buffered saline, followed by adding 900 μl fresh medium and 100 μl of neat DMSO, 100 mM 1M7 in DMSO, or 250 mM 5NIA in DMSO. After immediate mixing, cells were incubated at 37°C for 5 min. Then cells were harvested, and RNA was purified using RNeasy Mini Kit (Qiagen). The mutational profiling (MaP) reverse transcription was carried out with SuperScript II reverse transcriptase (Life Technologies) in 1× MaP buffer (50 mM Tris (pH 8.0), 75 mM KCl, 6 mM MnCl_2_, 10 mM DTT and 0.5 mM dNTPs), using random primers. The cDNAs were amplified by PCR using *Xist*-specific primers and Q5 high-fidelity DNA polymerase (NEB). The amplified DNA fragments were gel purified and pooled together. Construction of high-throughput sequencing libraries and sequencing were carried out by Novogene. The following primers were used for PCR amplification of *Xist* cDNA: *Xist*_-3 (F: 5′-TAGGCCATTTTAGCTATGACTGT-3′, R: 5′-TTTGAACTCCCAGACCTCTTC-3′); *Xist*_-1 (F: 5′-GAGACATGGTCTCATAAAGCC-3′, R: 5′-TGTGTGGAACCGAGGAAATA-3′); *Xist*_3 (F: 5′-AGGACTACTTAACGGGCTTA-3′, R: 5′-AGGGTAATCAATCACCTGCA-3′); *Xist*_4 (F: 5′-CCCAGCATCCCTTTCCATTTC-3′, R: 5′-AATTGCCAATGTGCTATGAG-3′); *Xist*_6 (F: 5′-GTCTCCTTGTGTTGTCTAATTCG-3′, R: 5′-TTCTGGACCTATTGGGAAGGG-3′); *Xist*_8 (F: 5′-ACAAAAAGCTTACAGGCCACA-3′, R: 5′-AATAGACACAAAGCAAGGAAG-3′); *Xist*_10 (F: 5′-TACTGAGGGTGATGAGTCTGT-3′, R: 5′-TCAGCAATGTCATATCAAACAC-3′); *Xist*_11 (F: 5′-TCCATTGACCACTTTTCTGAATCAC-3′, R: 5′-AAGATACTTGTCTTAAACATTCTGC-3′); *Xist*_12 (F: 5′-AGCAGAAAGAGGGTTGTACG-3′, R: 5′-TGATGGAATTGAGAAAGGGCAC-3′); *Xist*_14 (F: 5′-GGTTCCTACCACTATGCCCTG-3′, R: 5′-AAAACCCCATCCTTTATGCAA-3′); *Xist*_15 (F: 5′-TCACATGCTTTCTTATTTCAGCC-3′, R: 5′-AGTTAACACTGTGCACATTTAC-3′); *Xist*_16 (F: 5′-CCCATCTATACCCCCTCCAT-3′, R: 5′-GCAAGGGTAGTATTAGGACCTTGAG-3′); *Xist*_18 (F: 5′-CGTCTGATAGTGTGCTTTGC-3′, R: 5′-GGCTTGGGATAGGTCTGAAA-3′); *Xist*_19 (F: 5′-TGTTGGTGTTTGCTTGACTTCC-3′, R: 5′-AAACTTTAAGGACTCCAAAGTAAC-3′); *Xist*_20 (F: 5′-AGCGGACTGGATAAAAGCAAC-3′, R: 5′-CATCACAGTCTAATTCCATCCTG-3′); *Xist*_21 (F: 5′-GCTTGGTGGATGGAAATATGG-3′, R: 5′-CGTTATACCGCACCAAGAAC-3′).

### RNA FISH, immunostaining and immuno-RNA FISH

RNA FISH, immunostaining and immuno-RNA FISH were carried out as previously described (16). Immunostaining for H3K27me3 was performed using a mouse monoclonal antibody (Abcam; ab6002; 1:500) with a secondary antibody conjugated with Alexa-647 (ThermoFisher; A-21236; 1:1000). Immunostaining for H2AK119ub was performed using a rabbit monoclonal antibody (Cell Signaling Technology; D27C4; 1:2000) with a secondary antibody conjugated with Alexa-647 (Abcam; ab150075; 1:1000). Immunostaining was followed by RNA FISH. The *Xist* RNA was detected with Sx9 probe, a P1 DNA construct containing a 40 kb genomic fragment covering the *Xist* gene. The *Usp9x* probe was prepared with a BAC DNA (RP24-306P3). Nucleotide analogs used in probe labeling were Cy3-dUTP (Amersham, Cat# PA53022).

### Plasmid constructs

A mouse *Ssb* cDNA clone was purchased from OriGene (MG206549) and the sequence of the cDNA was confirmed by Sanger sequencing (data not shown). A series of in-frame deletions of various functional domains of *Ssb* were generated by PCR amplification using Herculase II Fusion Enzyme (Agilent Technologies) followed by Gibson Assembly (NEB). The sequences of the cloned cDNA fragments were confirmed by sequencing (data not shown).

For rescue experiments, the plasmid constructs were transfected into a female 3F1 ES cells with *Ssb* stably knockdown. G418 (ThermoFisher) was used at 250 μg/ml to select for stably transfected cells. Single surviving colonies were picked and examined using RT-qPCR. The shRNA construct, which worked efficiently against *Ssb*, was designed against the LAM domain, but not against the 3′ UTR of the RNA. Therefore, the shRNA is against some of the rescue plasmid constructs, except ΔLAM. Nonetheless, transfecting the *Ssb* knockdown cells with the rescue plasmids should compensate the effect of *Ssb* knockdown and serve as a rescue assay to study the functional domains of La.

### Microscopy and live-cell imaging

Wide-field fluorescent microscopy work was carried out on an Eclipse Ti microscope (Nikon) with a digital camera (Clara Series model C01, Andor).

Live-cell imaging was performed on a CorrSight spinning disk confocal system (FEI Company) equipped with an Orca R2 CCD camera (Hamamatsu). 1 day before imaging, 800K feeder-free ES cells were seeded on fibronectin-coated glass-bottom dishes (MatTec Corp). Prior to live-cell imaging, cells were washed with 1× PBS and replaced with imaging medium composing complete medium for differentiating ES cells with DMEM substituted with FluoroBrite DMEM (ThermoFisher). 1 μg/ml of doxycycline was supplemented to the imaging medium to induce *Xist* expression. For live-cell time-lapse video recording, cells were placed into the microscope cell culture chamber heated to 37°C at least 1 hr before imaging. Imaging was carried out in a closed chamber maintained at 37°C with 5% CO_2_ and 90% humidity. A 488-nm laser line (iChrome MLE-LFA) was set at 100% laser power. Images were acquired using a PlanApo 63×/1.4 N.A. oil-immersion objective (Zeiss) (heated to 37°C) with standard filter sets. The exposure time was set at 200 ms. All live-cell time-lapse video recording, unless explicitly stated otherwise, was carried out in a 2-hr time span with a 2-min time interval. For each time point, a 10-μm thick Z-stack with a 1-μm interval was collected. Autofocus system (Focus Clamp) was used to minimize out-of-focus throughout the recordings. Time-lapse imaging was started 1 h after the addition of doxycycline for differentiating cells. All acquired images were processed and analyzed using ImageJ (11). Drift correction was applied to all time-lapse recordings.

In the ‘sunset’ experiments, the snap-shot images of *Xist* signals in live cells were captured with an 800-ms exposure time at 100% laser power in 10-μm Z-stacks at 1-μm intervals.

### Data analysis

For padlock SNP capture, we only selected the SNPs which were allelotyped in all six samples. A nucleotide position with a read count less than 10 was considered as undetected and its read count was set to 0. Read counts of all the SNPs from one gene were combined to calculate the allelotype of the gene. Genes known to escape XCI were removed. The allelotype of each gene is calculated as Log ((129 count + 10)/(cast count + 10)). To avoid division by 0, we added a pseudocount of 10 to each read count. The sequencing data is available in sequence read archive (SRA, accession number PRJNA545157).

For ATAC-seq, in a small-scale pilot data analysis, we mapped 10 million reads from each sample onto the mouse genome (mm9) using bowtie2 (17). The sequencing depth of each sample was normalized to the total number of reads with a single best alignment position along the mouse genome outside of chromosome X. The alignment position of each read indicates the site of a transposon insertion event. The number of insertion sites along a given 1 mb region was counted and named as the ‘Cut Count’ of the region. The sequencing data is available in SRA (accession number PRJNA545157). The results of this pilot analysis were shown in Supplementary Figure S5B. All the sequencing data was used to generate the rest of the ATAC-seq results. The data analysis procedure is similar. In brief, sequencing reads were aligned through Bowtie2, reads without a single best alignment position were filtered out. PCR duplicates were then removed by Picard (http://broadinstitute.github.io/picard/). Genome feature annotation was done using ‘annotatePeak’ from ChIPseeker package (18). The sequencing data is available in SRA (accession number PRJNA545157).

For SHAPE-MaP, the data analysis software ‘ShapeMapper_v1.2’ was downloaded from Dr Kevin Weeks lab website (https://weeks.chem.unc.edu/software.html) (19). The recommended sample data set ‘shapemapper_example_data’ (https://weeks.chem.unc.edu/software.html) was also downloaded for software test runs. The source code written in Python 2 was revised for Python 3 updates. The source code was also modified to calculate SHAPE reactivity using one control (MutationRate^1M7^ – MutationRate^DMSO^) instead of two controls ((MutationRate^1M7^ – MutationRate^DMSO^)/MutationRate^Denatured^). The following parameters were customly adjusted for the data analysis of this study: primerLength = 30; minDepth = 5000; trimBothEnds = on. The rest parameters were kept the same as the default setting of the software. The software was used for counting mutation and generating the parameters used for RNA structure analysis. Other data analysis, such as profiled nucleotides, mutation rate profiles and principle component analysis, was performed using Perl and Python scripts built in-house. The in-cell SHAPE sequencing data is available in SRA (accession number PRJNA589511 for 1M7 data; accession number PRJNA756708 for 5NIA data).

## RESULTS

### ‘FLAG-out’ the *Xist*-binding proteins

After *Xist* induction, we fixed the differentiating mouse ES cells by UV-crosslinking and performed FLAG affinity pull-down (Figure 1B). Quantitative RT-PCR revealed that around 75-fold enrichment of *Xist* RNA was achieved by FLAG immunoprecipitation (Supplementary Figure S1). Mass-spec was used to analyze two protein pull-down samples, a Dox-treated i-FLAG-*Xist* sample and a Dox-treated i-Empty sample (Figure 1B). Sixty nine proteins were identified only in i-FLAG-*Xist* but not in i-Empty (Supplementary Table S2A–C). Furthermore, for the proteins identified in both samples, we ranked them according to their protein scores in each sample and calculated the ranking gains. hnRNPM, a known *Xist*-binding protein, showed a ranking gain of 11 in i-FLAG-*Xist*. Therefore, additional 69 proteins with ranking gains higher or equal to 11 were also selected as candidate proteins (Supplementary Table S2D). In total, 138 candidate proteins were selected (Figure 1C and Table S2). Among the selected candidates, nine proteins were also identified as *Xist*-binding proteins in previous studies (2–4). Interestingly, the 138 candidate proteins can be clearly classified into six functional groups: (i) nuclear actin and related proteins (38 proteins); (ii) chromatin and related proteins (13 proteins); (iii) DNA and RNA binding proteins (20 proteins); (iv) nuclear RNP, ribosomal and nucleolar proteins (30 proteins); (v) membrane proteins (8 proteins); (vi) other proteins (29 proteins) (Figure 1C and D, and Table S1). The complete list of the candidate proteins is shown in Table S1. Selected candidate proteins are shown in Figure 1D.

### Validation of the functional significance of Ssb in the induced XCI

From the candidate proteins, we shortlisted three proteins for individual validation. Myb-binding protein 1A (Mybbp1a, Q7TPV4) and TAR DNA-binding protein 43 (Tardbp, Q921F2) were selected because they are known transcription repressors (20,21). The Lupus autoantigen La (P32067, encoding-gene name: *Ssb*) was selected because systemic lupus erythematosus (SLE) is an autoimmune disease characterized by a strikingly high female to male ratios of 9:1 (22). Moreover, its autoimmune antigen Ssb is a ubiquitous and versatile RNA-binding protein and a known RNA chaperone (23). All the three selected candidates have also been identified as *Xist*-binding proteins in previous studies (2,4). Moreover, the knockout of these three genes all lead to early embryonic death. *Tardbp* knockout causes embryonic lethality at the blastocyst implantation stage (24). *Mybbp1a* and *Ssb* knockout affect blastocyst formation (25,26). Early embryonic lethality is a mutant phenotype consistent with a critical role of the mutated gene in XCI (1).

**Figure 2.**
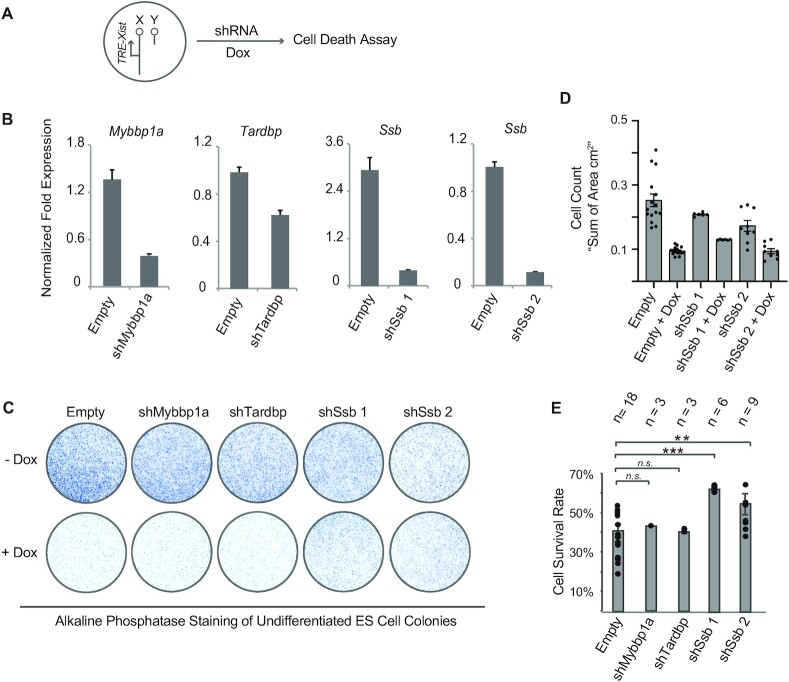
Individual validation of three candidate *Xist*-binding proteins. (**A**) The experimental design of the cell survival assay. (**B**) Quantitative RT-PCR to assess the effect of shRNA knockdown. Clonal mouse ES cell lines stably transfected with the shRNA constructs were established. Cells stably transfected with the empty shRNA vector (Empty) serve as the control. Data are shown in relative fold expression. Normalization was performed using *Actb* and *Gapdh*. Error bars indicate SEM (*n* = 3). (**C**) Representative macrographs of alkaline phosphatase staining on undifferentiated mouse ES cell colonies, using EGFP-iXist cells (10). Clonal ES cell lines stably transfected with the shRNA constructs against *Mybbp1a*, *Tardbp*, *Ssb* and the empty shRNA vector were cultured as undifferentiated ES cells. Dox treatment was carried out for 5 days. (**D**) Cell count was quantified as the sum of area of alkaline phosphatase staining. Data are shown as mean ± SEM. *n* = 15 for empty; *n* = 6 for *shSsb*1; *n* = 9 for *shSsb*2, from at least two independent experiments. (**E**) Cell survival rate, defined as the ratio of cell numbers with and without Dox (+Dox/–Dox), was calculated by alkaline phosphatase-stained colony counts. Data are shown as mean ± SEM. The statistical analysis used is the Student's *t*-test. **P < 0.01; ***P < 0.001; *n* = the number of experimental replications.

As the gene knockout of all three candidate genes causes early embryonic lethality, we chose to knock down their expression using shRNAs to validate the candidate genes’ roles in the induced XCI. The selected cell line is a male mouse ES cell line carrying an inducible X-linked *Xist* transgene. Inducible *Xist* expression in this cell line causes cell death due to the inactivation of the single X chromosome in male cells. About 60% of cells die after 4–5 days of doxycycline (Dox) treatment, which can be used as a convenient assay to assess the functionalities of XCI (Figure 2A). We established clonal ES cell lines stably expressing the shRNAs and confirmed the shRNA knockdown efficiencies (Figure 2 A and B). In the absence of Dox, knocking down *Ssb* showed a negative effect on the cell growth rate (Figure 2C and D), consistent with the role of Ssb as a ubiquitous RNA-binding protein involved in house-keeping functions such as tRNA biogenesis. To avoid the interference of the altered growth rate caused by *Ssb* knockdown, the cell survival rate, defined as the ratio of cell numbers with and without Dox (+Dox/–Dox), was calculated to quantitatively assess XCI (Figure 2E). The cell survival rates of *Ssb* knockdown ES cell lines are increased from 40% to 60%, indicating that knocking down *Ssb* significantly rescued the cells from the toxicity of induced XCI (Figure 2C–E). These results further suggest that Ssb is involved in induced XCI. In the foregoing experiments, cells were cultured as undifferentiated ES cells during the induction. We performed the Dox-induced cell death assay in differentiating ES cells and obtained consistent results (Supplementary Figure S2). We further validated the interaction between Ssb and *Xist* RNA by RNA immunoprecipitation (RIP) and UV-crosslinking and immunoprecipitation (CLIP) (Supplementary Figure S3). Even though RIP and CLIP were performed with *Xist* inducible male ES cells ectopically expressing FLAG-Ssb, the overall expression level of *Ssb* mRNA in these cells is not higher than that in control cells (Supplementary Figure S3B), implying that the level of exogenous FLAG-Ssb is negligible in comparison with endogenous Ssb. These results confirm that Ssb is involved in the induced XCI in the male transgenic cell line.

Meanwhile, our results do not confirm the functional roles of Mybbp1a and Tardbp in XCI (Figure 2C and E). However, it is possible that the knockdown efficiencies of these two genes were not high enough to overcome the thresholds required to disturb XCI. Alternatively, there might be some redundant factors for Mybbp1a and Tardbp that compensate the loss of these two factors.

### Validation of the functionality of Ssb in endogenous XCI in female cells

**Figure 3.**
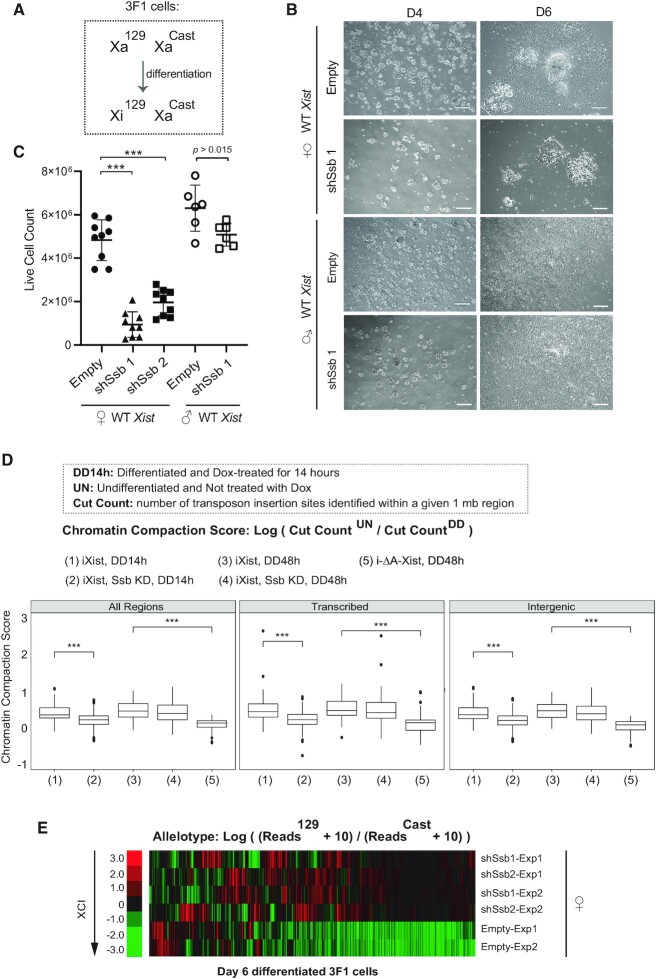
Functional validation of Ssb in female mosue ES cells during the endogenous XCI. (**A**) The genotype and the preemptive XCI choice phenotype of 3F1 female mouse ES cells. (**B**) Representative brightfield microscope images showing the differentiating 3F1 ES cells at day 4 (D4) and day 6 (D6) of *in vitro* differentiation. Scale bars, 250 μm. (**C**) Live cell number counts on *in vitro* differentiation day 6. Data are shown as mean ± SEM. The statistical analysis used is the Student's *t*-test. ***P < 0.001; n = 9 from three independent experiments. (**D**) Box plots of chromatin compaction scores of the ATAC-seq assay. The mouse X chromosome is divided into ∼160 1-mb regions. The Cut Count of each region was analyzed, and the chromatin compaction scores of each 1Mb region was quantitatively calculated. For detailed analysis, the genomic regions are further categorized as intergenic and transcribed regions. i-ΔA-*Xist* is an inducible mutant *Xist* transgene, in which the critical A-repeat region is deleted. ***P < 0.001. (**E**) Heatmaps of chromosome-wide RNA allelotyping results. RNA samples were isolated from differentiating ES cells on day 6 of *in vitro* differentiation.

To validate the functionality of Ssb in endogenous XCI, we performed shRNA knockdown of *Ssb* in female mouse ES cells (Figure 3A). We confirmed the knockdown efficiency on the selected clonal cell lines stably expressing shRNAs against *Ssb* (Supplementary Figure S4). XCI can be triggered in female mouse ES cells by *in vitro* differentiation. Massive cell death usually occurs during this process in female mouse ES cells with XCI defects (1). Consistent with this notion, we observed significant cell death in the *Ssb* knockdown cells during *in vitro* differentiation (Figure 3B and C). Importantly, on the *Ssb* knockdown background, significantly more cell death was observed in female cells than male cells (Figure 3B and C). Although *Ssb* knockdown showed negative effect on the cell growth rate of male ES cells (Figure 2C and D), the morphology of male embryoid bodies (EBs) on day 4 of *in vitro* differentiation were comparable to the wild type EBs. The EBs could attach to the surface of the tissue culture flask and showed significant amount of expansion two days later (Figure 3B). This is in clear contrast to the female EBs. With *Ssb* knockdown, the female EBs were small and unhealthy on day 4 of *in vitro* differentiation. The EBs barely attached to the surface of the tissue culture flask and showed no or very limited amount of expansion two days later (Figure 3B). These results support a functional role of Ssb in XCI.

As Ssb is an RNA-binding protein involved in various house-keeping functions, the massive cell death, which occurred in *Ssb* knockdown female mouse ES cells during *in vitro* differentiation, may not be directly and solely attributed to XCI defects. Therefore, we performed ATAC-seq (Assay for Transposase-Accessible Chromatin with high-throughput sequencing) to examine the chromatin compaction along X chromosome. We also performed padlock SNP capture to directly assess the XCI status by allelotyping the X-linked gene expression chromosome-wide.

ATAC-seq was performed using male mouse ES cell lines carrying an X-linked inducible *Xist* transgene so that the sequencing reads mapped onto X chromosome only reflect the chromatin status of one X chromosome, the inactive X. In ATAC-seq, the density of transposon insertion reveals the status of chromatin compaction. We analyzed the transposon insertion sites genome-wide and defined ‘Cut Count’ as the number of transposon insertion sites identified within a 1-Mb region. The chromatin compaction status during the induced XCI was then quantitatively measured by the ratio of the Cut Counts between the uninduced undifferentiated cells and the differentiated cells treated with Dox (Figure 3D). Induced XCI clearly caused chromatin compaction in wild type cells, meanwhile the induced expression of a mutant *Xist* transgene with the critical A-repeat region deleted (i-ΔA-*Xist*) failed to generate chromatin compaction (Figure 3D, Supplementary Figure S5, and Supplementary Table S3). These results serve as controls to validate the experimental system. On the *Ssb* knockdown background, induced XCI also caused chromatin compaction, but to a lesser degree than that in wild type cells (Figure 3D and Supplementary Figure S5). This observation was clearly made after 14 h Dox treatment. After 48 h Dox treatment, the chromatin compaction status was comparable between the wild type sample and the *Ssb* knockdown sample, although a slightly lesser degree of chromatin compaction was still visible in the knockdown sample especially for the transcribed regions (Figure 3D and Supplementary Figure S5). These results directly connect Ssb to the heterochromatinization of Xi, supporting a functional role of Ssb in XCI. Meanwhile, the results also show that the *Ssb* knockdown cells encountered difficulties, such as chromatin compaction, during the early onset of XCI. We further discuss this issue in the later sections.

To directly assess the XCI status of X-linked genes, we comprehensively allelotyped X-linked genes. The shRNA knockdown was performed on a female ES cell line 3F1 (27), which carries Xs from two genetic backgrounds, the 129 mouse strain (X^129^) and the *Mus musculus castaneus* (CAST/Ei) mouse strain (X^Cast^). The ‘preemptive choice’ mutant phenotype of 3F1 cells causes non-random inactivation of the X^129^ allele (27) (Figure 3A). Therefore, the XCI status of an X-linked gene can be evaluated by RNA allelotyping of X^129^ and X^Cast^, which provide ample choice of single nucleotide polymorphisms (SNPs). Padlock SNP capture, a high-throughput and high-resolution RNA allelotyping method (12), was performed to profile the XCI status of X linked genes chromosome-wide. The padlock probe library was designed to target 2969 SNPs covering 1110 (∼55%) of the X-linked genes (12). 457 X-linked genes were successfully allelotyped in the experiment. Padlock SNP capture detected bi-allelic expression of genes along the entire X chromosome in *Ssb* knockdown cells, demonstrating obvious XCI defects (Figure 3E and Supplementary Table S4). This result provides the direct evidence confirming the critical functionality of Ssb in XCI.

### Knockdown of *Ssb* impairs *Xist* cloud formation

**Figure 4.**
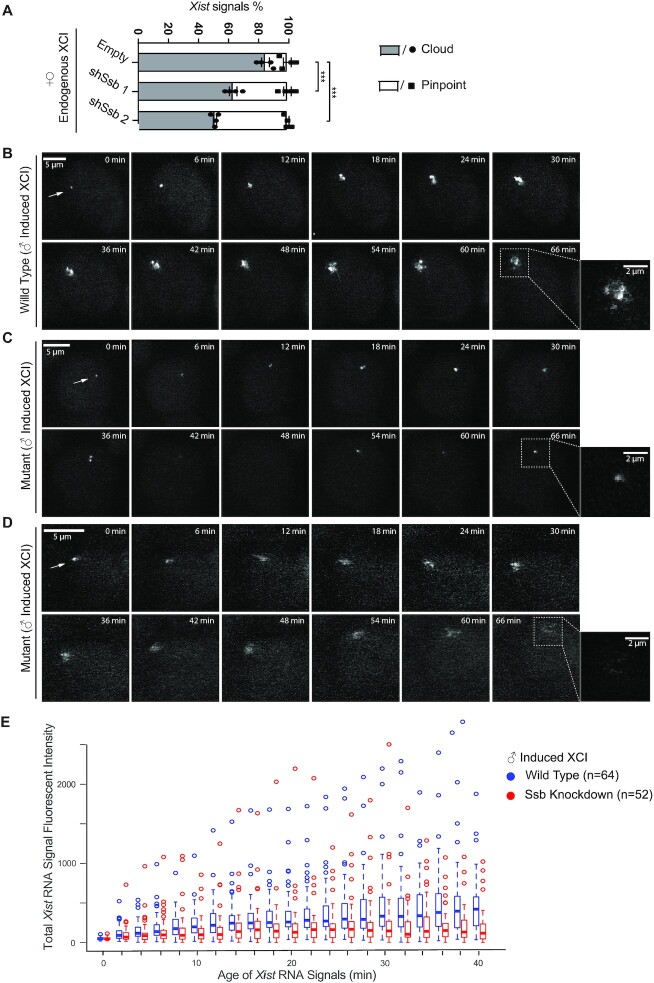
Ssb plays critical roles in *Xist* cloud formation. (**A**) *Xist* RNA FISH results of day 6 *in vitro* differentiation female mouse ES cells. Quantification of *Xist* RNA signals from four independent experiments is shown. Data are shown as mean ± SEM. The statistical analysis used is *X^2^* test. *** *P* < 0.001; *n* = 505 for empty; *n* = 540 for *shSsb* 1; *n* = 499 for *shSsb* 2. (**B–D**) Live-cell imaging of *Xist*. The *Xist* RNA cloud formation processes in one representative wild type cell and two *Ssb* knockdown cells are shown. Dox treatment was started after cells were *in vitro* differentiated for 16 h. Live-cell imaging was started 1 h after Dox treatment. All the live-cell imaging was carried out in a 2-h time span with a time interval of 2 min. For direct comparison between the two *Xist* signals, images are shown in a 66 min time span with a 6 min time interval. The time point when a signal was first detected is defined as time zero for the signal. Maximum intensity z-projections are shown. (**E**) The total fluorescent intensity of an *Xist* RNA signal in live cells was measured every 2 min for the first 40 min after the signal first appeared. The measurement was done on 64 *Xist* signals from wild type cells and 52 *Xist* signals from *Ssb* knockdown cells. *Xist* signals were randomly selected from two independent experiments. The data are presented in box plots. Student's *t-*test was performed to compare the pair of data sets at each time point. *P* > 0.05 for the time points of 0–8 min; *P* < 0.05 for the time points of 14 and 16 min; *P* < 0.009 for the rest of the time points.

To further study the functional roles of Ssb in the endogenous XCI, we investigated the *Xist* cloud formation in *Ssb* knockdown cells. In undifferentiated female ES cells, *Xist* expression is detected as a pinpoint signal associated with its gene locus in *cis*. Upon differentiation, *Xist* expression is up-regulated along the chosen Xi and *Xist* RNA transcripts spread out to cover the chromosome territory *in cis*. Thus, in differentiated female cells*, Xist* expression is detected as a cloud signal (the *Xist* cloud) enveloping the Xi chromosome territory. We performed *Xist* RNA FISH in differentiating ES cells. After 6 days of *in vitro* differentiation, *Xist* clouds could be detected in >80% of the wild type cells. However, in *Ssb* knockdown cells, *Xist* clouds were detected in a significant lower percentage of cells (Figure 4A and Supplementary Figure S6). Given that significant cell death occurs in *Ssb* knockdown cells during *in vitro* differentiation and RNA FISH cannot be applied to dead cells, the percentage of cells showing faulty *Xist* cloud formation should be even higher in *Ssb* knockdown cells. Taken together, these results show that Ssb is involved in *Xist* cloud formation.

To avoid the shortcomings of RNA FISH not applicable to dead cells, and to observe the process of *Xist* cloud formation during the early onset of XCI, we performed live-cell imaging of *Xist* RNA. We previously established a live-cell imaging system of the *Xist* RNA in a transgenic male mouse ES cell line carrying an X-linked single-copy inducible *Xist* transgene with PBSb sites fused to its 5′-ends (Figure 1A). The cell line also stably expresses a PUFb-GFP fusion protein. Therefore, *Xist* RNA can be efficiently GFP-tagged in live cells. We chose an inducible transgene to study the *Xist* RNA in live cells, because the system provides a synchronized onset of XCI in the cell population. In female cells, during the *in vitro* differentiation, the onset of the endogenous XCI occurs in an unsynchronized manner. For a given cell, the onset of XCI may occur at any time during a window of a few days. However, high-quality live-cell imaging can only be performed within a 2-h time window due to technical issues, such as photo-bleaching and phototoxicity. In our experimental system, Dox treatment was started after the cells were *in vitro* differentiated for 16 h. Live-cell imaging was performed 1 h after Dox treatment and lasted for 2 h with a 2-min time interval. Because the selected time window belongs to the early onset stage of XCI, the issue of cell death caused by XCI defects is circumvented. In wild type differentiating cells, the behavior of *Xist* cloud formation is synchronized and consistent (10). The *Xist* RNA signals first appear as small pinpoint signals that then gradually grew into ∼2 μm large *Xist* RNA clouds within 60–90 min (Figure 4B, and Supplementary Movies S1). In *Ssb* knockdown cells, the *Xist* cloud formation clearly encountered difficulties (Figure 4C–E, and Supplementary Movies S2, S3). In some cells, an *Xist* RNA signal first appeared as a small pinpoint signal, but the pinpoint signal failed to stabilize. Instead of growing into a large cloud signal, the pinpoint signal was on and off during the first 60–90 min after the signal first appeared (Figure 4C, and Supplementary Movies S2). In some other cells, a pinpoint *Xist* RNA signal grew into a faint cloud signal, and the cloud signal quickly diffused and vanished (Figure 4D, and Supplementary Movies S3). We analyzed 64 *Xist* signals in wild type cells and 52 *Xist* signals in *Ssb* knockdown cells. The total fluorescent intensity of each *Xist* signal was measured every 2 min during the first 40 min after the signal first appeared (Figure 4E). The results clearly revealed the faults of *Xist* cloud formation on the *Ssb* knockdown background. Quantitative RT-PCR showed that *Ssb* depletion does not change *Xist* induction in the *Xist* inducible male ES cell line, thus excluding the possibility that *Ssb* depletion affects *Xist* cloud formation by down-regulating *Xist* RNA (Supplementary Figure S7). In summary, the observed behaviors of *Xist* cloud formation in *Ssb* knockdown cells are heterogenous but consistently show the difficulties encountered in forming the *Xist* cloud. In *Ssb* knockdown cells, the *Xist* RNA transcripts failed to spread out and coat the X chromosome territory. It is likely that the RNA transcripts are quickly diffused and/or degraded. As *Xist* cloud formation is the initial step which triggers XCI, the defective *Xist* cloud formation should be the primary reason behind other XCI defects observed in *Ssb* knockdown cells. These results confirm that the defining XCI defect in *Ssb* knockdown cells is faulty *Xist* cloud formation.

### Knockdown of *Ssb* compromises the enrichment of Polycomb marks on Xi

**Figure 5.**
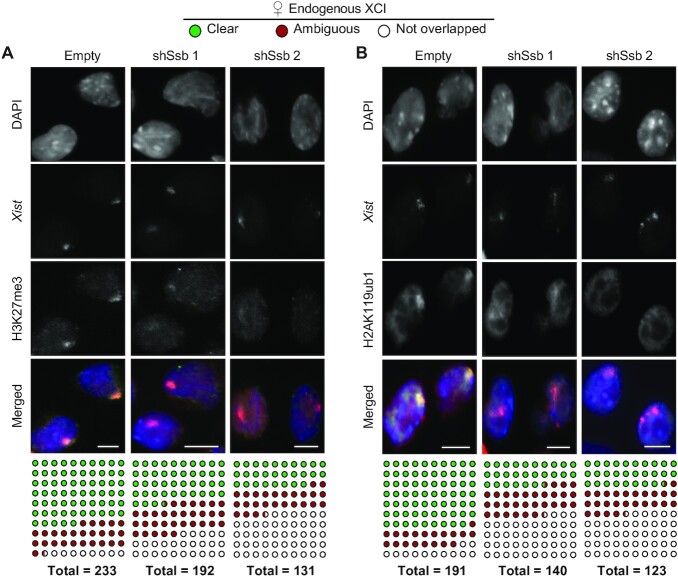
*Ssb* is involved in Polycomb mark enrichment along Xi. **(A and B)** Immuno-RNA FISH detecting H3K27me3 (**A**), H2AK119ub (**B**) and *Xist*. Female mouse ES cells were *in vitro* differentiated for 6 days. Immunostains were performed before the RNA FISH. DNA was counter stained with DAPI (blue). Scale bars, 8 μm. The total number of *Xist* clouds with clear, ambiguous or undetectable overlapping with the histone mark enrichment were tallied and tabulated below. *P* < 0.001, from all chi-square tests comparing *Xist* cloud counts between a control sample and an *Ssb* knockdown sample.

Even though *Xist* cloud formation is less efficient in the *Ssb* knockdown cells, the knockdown cell population behave heterogeneously and *Xist* clouds are formed in a substantial fraction of surviving cells. We further investigated whether Ssb regulates other events during the later stages of XCI. It is known that, in order to inactivate one large chromosome, *Xist* cloud recruits other silencing factors to heterochromatinize Xi with multiple layers of epigenetic modifications (1). Two histone modifications enriched along Xi are the Polycomb marks, H3K27me3 and H2AK119ub (9). We performed immuno-RNA FISH to study the role of Ssb in Polycomb marks enrichment along Xi. After 6 days *in vitro* differentiation, most of the *Xist* clouds signals in RNA FISH overlapped with enrichments of Polycomb marks detected by the immunostains (Figure 5). However, the enrichment of Polycomb marks along Xi is significantly disrupted in *Ssb* knockdown cells. These results suggest that Ssb is also involved in establishing the enrichment of Polycomb marks along Xi.

### The RNA chaperone domain of Ssb is critical in XCI

**Figure 6.**
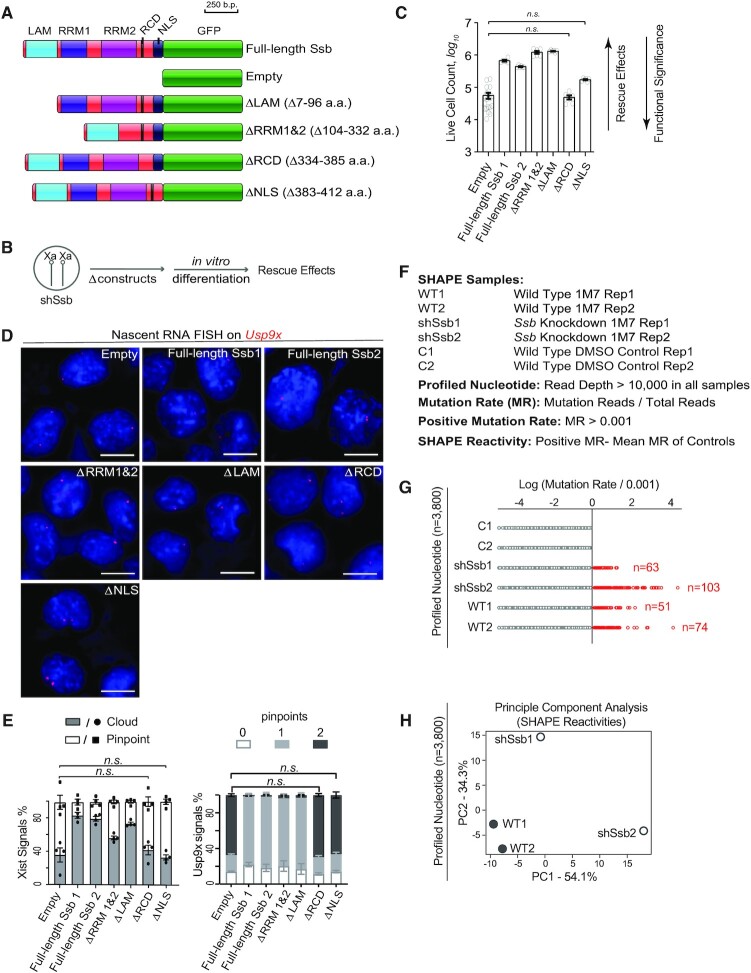
Dissecting the functional domains of Ssb in XCI. (**A**) Diagram of the functional domains of Ssb and the serial of plasmid constructs carrying in-frame deletions of selected functional domains (Δ constructs). (**B**) Experimental design of the rescue experiment. (**C**) The rescue effects of Δ constructs on cell survival of *Ssb* knockdown cells during *in vitro* differentiation. Cell counts of day 6 *in vitro* differentiation are shown. Data are shown as mean ± SEM. The statistical analysis used is the Student's *t*-test. Samples that do not significantly differ from empty vector (*P* > 0.05) are indicated as n.s.. *P* < 0.0001 for all the rest of the pairwise comparison between Empty and any one of the serial Δ constructs. *n* = 18 from 6 independent experiments for empty; *n* = 6 from two independent experiments for the rest of the constructs. (**D**) Representative images of RNA FISH on *Usp9x* nascent transcripts. (**E**) Quantification of *Xist* and *Usp9x* RNA FISH signals. Data are shown as mean ± SEM (*n* > 230 for all the constructs). Samples that do not significantly differ from empty vector (*P* > 0.05) are indicated as n.s.. *P* < 0.001 for all the rest of the pairwise comparison between Empty and any one of the serial Δ constructs. The statistical analysis used is *X^2^* test. (**F**) Sample identities of SHAPE experiments using 1M7 as probe and parameters used in data analysis. (**G**) Mutation rates of the 3800 profiled nucleotides of each sample. (**H**) A PCA plot of the SHAPE reactivity profiles using 1M7 as probe.

Ssb is an RNA-binding protein with 5 defined functional domains including three RNA-binding domains (La motif, LAM; RNA recognition motif 1, RRM1; RNA recognition motif 2, RRM2), an RNA chaperone domain (RCD) and a nuclear localization signal (NLS) (28,29). We generated plasmid constructs carrying in-frame deletions of the selected domains of Ssb (Figure 6A) and stably transfected the *Ssb* knockdown cells with these ‘rescue’ plasmid constructs (Figure 6B). GFP fusion was included in the plasmid design for checking transfection efficiencies and the transgene expression levels in the selected clonal cell lines (Supplementary Figure S8). We first rescued female ES cells with a full-length *Ssb* construct. Indeed, the full-length *Ssb* construct showed significant rescue effects on cell survival, *Xist* cloud formation and Polycomb marks enrichment (Supplementary Figure S9). Meanwhile, all Δ constructs showed significant rescue effects of various degrees on the cell survival during *in vitro* differentiation, except for ΔRCD and ΔNLS, indicating the critical roles of these two functional domains (Figure 6C). For the silencing of a X-linked gene *Usp9x* and *Xist* cloud formation, ΔNLS and ΔRCD showed no rescue effect, while other Δ constructs showed significant rescue effects of various degrees (Figure 6D and E). These results show that the RCD and NLS are required for the functionality of Ssb in XCI. As XCI is a nuclear event, it is obvious why the NLS of Ssb is critical for its functionality in XCI. On the other hand, the critical role of RCD of Ssb in XCI is intriguing.

### *Xist* RNA is misfolded and less stable on *Ssb* knockdown background

We believe Ssb is an *Xist*-binding protein important for folding of the RNA, possibly as an RNA chaperone. To assess the folding of *Xist* RNA, we performed SHAPE-MaP (selective 2′-hydroxyl acylation analyzed by primer extension and mutational profiling) (19). The RNA structure was probed by1-methyl-7-nitroisatoic (1M7), which reacts with 2′-hydroxyl group and forms adducts along the RNA with a preference of unstructured regions. Chemical adducts along the treated RNA affect the fidelity of reverse transcription reaction and are detected as ‘mutation rates’ by sequencing, which can be used to assess the RNA structure. Using male ES cell lines carrying the inducible *Xist* transgene, we performed in-cell SHAPE experiments in wild type cells (duplicate samples), *Ssb* knockdown cells (duplicate samples) and DMSO control (wild type cells treated with DMSO, duplicate samples). Along the ∼18kb *Xist* RNA, we only analyzed the 3800 nucleotides which were covered by >10 000 reads in each sample (‘profiled nucleotides’, Figure 6F and Supplementary Table S5). We selected 0.1% mutation rate as the threshold. A mutation rate greater than 0.1% is considered ‘positive’. 0.1% is close to the error rate of the current Illumina sequencing technology and occurs to be a natural boundary separating the two control samples and the four 1M7-treated samples in our experiments. Among the profiled nucleotides, all the 291 incidents of positive mutation rate were detected in 1M7-treated samples (Figure 6G). The distribution patterns of the nucleotides with positive mutation rates are consistent between the two wild type samples. These data validate the SHAPE experimental system. Interestingly, principle component analysis of the global SHAPE reactivity profiles revealed that the distribution patterns of the wild type samples are clearly distinguishable from mutation samples; and the two mutant sample also different from each other (Figure 6H). This observation can be best illustrated by the 467 profiled nucleotides along the 5000^th^–6000^th^ nucleotide region of *Xist* (Supplementary Figure S10A). Consistently, SHAPE experiments with another chemical probe, 5-nitroisatoic anhydride (5NIA) also demonstrated that the folding of *Xist* RNA is altered in *Ssb* knockdown cells (Supplementary Figure S10B–D and Supplementary Table S6). These data show that *Xist* RNA is misfolded in *Ssb* knockdown cells. Very likely, the misfolded RNA forms a random pool of transcripts which lacks a consensus structure and shows poor consistency from sample to sample.

**Figure 7.**
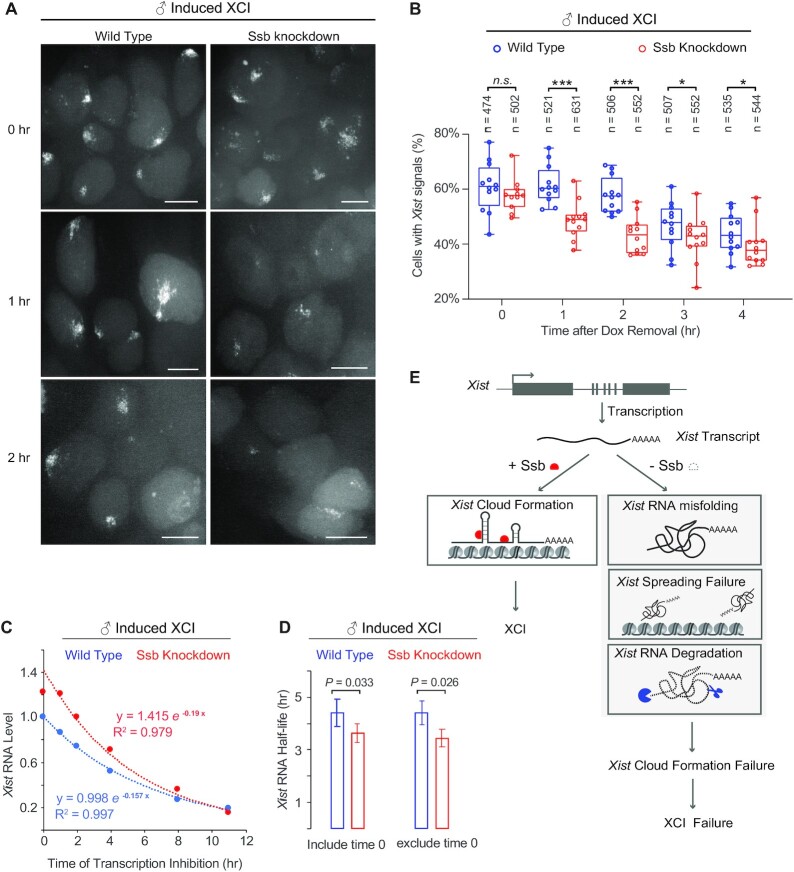
Stability of *Xist* on *Ssb* knockdown background by live-cell imaging after Dox removal, and a proposed model of how Ssb is involved in XCI as an *Xist*-binding RNA chaperone. (**A**, **B**) The disappearance of *Xist* cloud signals in live cells after Dox removal. Cells were treated with Dox overnight in differentiating conditions. *Xist* cloud signals were assessed hourly after Dox removal. Representative images are shown. Scale bars, 8 μm. At each time point, images were collected from ∼12 randomly selected fields. The percentage of cells with *Xist* clouds in each field was quantified and the results are shown in box plots. The total number of cells analyzed at each time point is shown on the top. The statistical analysis used is the Student's *t*-test. *P = 0.05; ***P < 0.01. (**C**, **D**) Measurement of *Xist* stability. Male ES cell lines carrying the inducible *Xist* transgene, with or without *Ssb* shRNA, were treated with Dox for 24 h. Dox was then removed, and Actinomycin D (5 μg/ml) was added (defined as 0 time point). Quantitative RT-PCR was performed to measure *Xist* RNA levels in samples collected at indicated time points. Normalization was performed using *Actb*. (**C**) A representative data was fitted using an exponential decay model with corresponding R-squared shown. The half-life of *Xist* was then calculated. (**D**) Calculated half-life of *Xist* from three independent experiments, with or without the time 0 point. Error bars indicate SD (*n* = 3). (**E**) The proposed model.

We further investigated whether the misfolded *Xist* transcripts are subjected to degradation. To assess the stability of *Xist* RNA on *Ssb* knockdown background, we studied the disappearance of the *Xist* cloud signals after Dox removal (the ‘sunset’ process). Cells were cultured in differentiating conditions and treated with Dox overnight before Dox removal. The rate of the sunset process reflects the stability of the *Xist* RNA. We observed a significantly faster sunset rate on *Ssb* knockdown background (Figure 7A and B), especially within the first 2 h after Dox removal. To further assess the stability of *Xist* RNA, we performed quantitative RT-PCR to quantify the *Xist* RNA during sunset. The cell lines used were male ES cell lines carrying the inducible *Xist* transgene (Figure 7C and D). *Xist* transcription was blocked by Dox removal and actinomycin D treatment. The data was best fitted using a first-order exponential decay model (Figure 7C). Half-life of *Xist* RNA was then calculated (Figure 7D). Consistent with our live-cell imaging data, quantitative RT-PCR results show that the half-life of *Xist* is significantly shorter on *Ssb* knockdown background. Interestingly, it seems the decay reaction kinetics of *Xist* in *Ssb* knockdown cells can be separated into two phases (Figure 7C). The first hour is the first phase of the reaction, which is a short phase and only contains two data points. The rest of the data points form the second phase of the decay reaction and can be better fitted in one reaction curve separately from the 0 time point. The half-life of *Xist* RNA estimated from the data of the second phase is shorter and with more statistical significance (Figure 7D). We provide our interpretation on this in the discussion section.

Taken together, SHAPE results show that the *Xist* RNA transcripts are misfolded on *Ssb* knockdown background, and the shorter half-life of *Xist* RNA suggests that the misfolded *Xist* RNA transcripts are subjected to faster degradation.

## DISCUSSION

Here, we show that FLAG-out is a useful method for profiling the interactome of lncRNAs. The advantage of FLAG-out is that once the target RNA is FLAG-tagged, the high specificity and high sensitivity of anti-FLAG antibody can be applied for the subsequent protein isolation. It should be noticed that selection of the PBS insertion site along the target RNA affects the outcome of FLAG-out. In this study, the PBSb sites are fused at the 5′ end of *Xist*, a position close to the most critical functional domain of *Xist*. In our previous study, we have shown that the 5′ PBSb fusion does not affect the *Xist* functionality (10). Given that 5′ PBSb fusion does not affect the *Xist* functionality, we believe that the fusion of PBSb to *Xist* and PUFb-FLAG binding may not alter the RNA’s structure and its association with protein partners. It is notable that only 9 out of 138 proteins identified by FLAG-out were identified as *Xist*-binding proteins in previous studies (2–4). In fact, *Xist* binding proteins identified in other studies did not have a good overlap either. It is very likely that none of these studies, including ours, comprehensively identified *Xist* binding proteins. A variety of technical issues limit the precision and sensitivity of the current methods.

Ssb is known as an RNA chaperone (29). Based on the results of this study, we propose that Ssb is an *Xist*-binding protein involved in *Xist* RNA folding and spreading (Figure 7E). In the *Ssb* knockdown cells, the misfolded *Xist* RNA transcripts are unstable and unable to spread and form the *Xist* clouds. These defects eventually lead to failure of *Xist* cloud formation and collapse of XCI. However, we still cannot rule out the possibility that the indirect effect of Ssb regulates XCI. Ssb might be required for the proper expression of a specific factor in the process of XCI. Without this specific factor, *Xist* RNA unfolds and degrades, leading to a defect in *Xist* cloud formation.

Spen, the critical *Xist*-binding protein identified in previous studies, only showed the earliest mutant phenotype around embryonic day E12.5 in the gene knockout animals (30,31). No female-specific phenotype was observed. These results are in contrast to the protein's critical roles in XCI, which is an early embryonic event occurring at the preimplantation stages of female embryos. This is why we selected three candidate genes with early embryonic lethality in their gene knockout animals for further validation. Because of early embryonic lethality, we chose to knock down the genes’ expression instead of knocking out the genes. Because the knockdown levels may vary among individual cells, the XCI process may be completed in some of the surviving cells especially at the late time points of the experiments. This explains why some of the XCI defects were more clearly observed in the knockdown cells at early time points, for example, the chromatin compaction defects (Figure 3D and Supplementary Figure S5). Nonetheless, the female-specific cell death occurred during in *vitro* differentiation of ES cells, the live-cell imaging results and many other experimental results of this study clearly reveal the critical roles of Ssb in XCI.

Ssb is an autoimmune antigen found in the serum of patients with autoimmune diseases. More than 80% of the patients with autoimmune disorders are female (22). It is a possibility that XCI is related to the striking sex ratio distortion of autoimmune disorders. Multiple hypotheses have been developed. As numerous X-linked genes are related to immune functions, dysregulated expression of these genes may cause malfunction of the immune system. Therefore, skewed XCI pattern (loss of mosaicism), imbalanced X-linked gene dosage (X chromosome reactivation in early T cell lineage and haploinsufficiency in X chromosome monosomy) have all been hypothesized as possible causes of the sex ratio distortion in autoimmune disorders (22,32). *Ssb* may be a potential genetic factor of autoimmune diseases. However, identifying Ssb, as a protein involved in XCI does not help to further explain the sex ratio distortion. Rather, we believe it is a consequence of autoimmune disorders. Nuclear RNPs are immunogenic (33). The inactive X chromosome coated with *Xist* RNA and its associated proteins is a large piece of nuclear RNP in female cells, which can trigger autoimmune responses when exposed to the immune system under abnormal situations.

Understanding the structure of lncRNAs is critical for understanding lncRNAs’ functionality. Initial efforts to elucidate the *Xist* RNA structure have been reported, but significant inconsistency exists among the results of the initial studies (14,34–36). Nonetheless, several interesting and important issues are revealed. First, the in-cell structure of the RNA is significantly different from the *ex-vivo* structure of the RNA purified with mild conditions. Second, subregions of *Xist* may form dynamic structures in living cells. SHAPE analysis suggests that the dynamic structures function as loading pads for protein recruitment (14). In PARIS, a method in which the RNA structures are fixed in cells and the interacting regions of RNAs (duplex sequences) are directly sequenced, significant amount of conflicting duplex sequences is detected, which shows the *Xist* structure is highly dynamic in living cells (35). In our study, identifying Ssb as a protein involved in *Xist* folding provides an important piece to the puzzle. Interestingly, Ssb possesses three RRMs, which may function in a synergistic manner in regulating *Xist* structure in living cells. The mechanistic details await future research. Here, we discuss three possible ways to imagine the mechanistic roles of Ssb in XCI. The three possibilities are not mutually exclusive to one another. Firstly, it is possible that Ssb may function as an *Xist* chaperone. Indeed, the RCD domain of the human Ssb protein has been characterized as an RNA chaperone domain (29). Ssb proteins have also been characterized as RNA chaperones in different biological events (28). Interestingly, it has been hypothesized that RNA chaperones may function as ‘capacitors’ to store and release the effect of genetic variation (28). Thus, the capacitor function of Ssb may help to explain the structural conservation of *Xist*, a lncRNA with poor sequence homology among mammalian species (1). Secondly, as Ssb is able to unwind RNA-RNA and RNA-DNA duplex, it may play a more active role in controlling the structural dynamics of *Xist* in living cells (23,37). These structural dynamics may be important for recruiting proteins onto the RNA and spreading of the RNA along its host chromosome territory. Thirdly, Ssb may be more directly involved in recruiting proteins onto *Xist*. Misfolding of *Xist* observed in *Ssb* knockdown cells may be a consequence of protein loading failures.

As a protein involved in tRNA maturation, a significant fraction of Ssb is located in the nucleolus (38,39). The nucleolus may function as a general factory for assembling nuclear RNP particles, not just for rRNAs. *Xist* and its associated proteins forms a unique RNP complex in female cells. The fact that Ssb is an *Xist*-binding RNA chaperone may be one reason behind the frequent association of the Xi with the nucleolus (16,40).

It is an interesting observation that the decay reaction of *Xist* in *Ssb* knockdown cells seems to consist of two phases. The first hour of the reaction is a transient phase in which the decay reaction shows a slow kinetics. The rest of the data points form the second phase of the reaction which shows a significantly faster kinetics. To interpret the data, we assume that the decay reaction of *Xist* is catalyzed by both endonucleases and exonucleases. Endonuclease creates nicks along the RNA, which further serve as the starting points for exonucleases. Without the RNA chaperone activity of Ssb, misfolded *Xist* RNA may be depleted for protein-binding and the RNA may be randomly folded in order to ‘protect’ the hydrophobic bases from the hydrophilic environment. Therefore, compared to the protein-binding biologically active form, the misfolded structures may be more compact, which prevents the initial endonuclease reaction. Once the endonuclease reaction generates enough nicks to trigger the action of exonucleases, the degradation reaction quickly speeds up. However, we do not have enough data to study the transient first phase in detail, therefore, this interpretation may be speculative at this point. The details of the decay reaction and, more importantly, the structural details of *Xist* await future studies.

## DATA AVAILABILITY

All data are available in the main text or the Supplementary Data.

## Supplementary Material

gkab1003_Supplemental_Files
