# Supplementary material for: The lupus autoantigen La/Ssb is an Xist-binding protein involved in Xist folding and cloud formation
Source: Nucleic Acids Res. 2021 Nov 1;49(20):11596–613. doi: 10.1093/nar/gkab1003 (PMC8599922; doi:10.1093/nar/gkab1003)
Supplement: gkab1003_Supplemental_Files [file gkab1003_supplemental_files.zip › Description of Table S2-6.docx]

**Table S2. *Xist* binding proteins identified by FLAG-out and mass spectrometry.**

(A) Proteins identified in the i-Empty sample. (B) Proteins identified in the i-FLAG-*Xist* sample. (C) Proteins identified in i-FLAG-*Xist*, but not in i-Empty. (D) Proteins identified in both i-Empty and i-FLAG-*Xist*.

**Table S3. The cut count and compaction score of ATAC-seq on chromosome 1 and X.**

Cut counts (A and C) and compaction scores (B and D) were calculated for regions on chromosome 1 (A and B) and Chromosme X (C and D). These data were presented in Fig. 3D and Fig. S5.

**Table S4. The allelotype score for individual SNPs detected by padlock SNP capture.**

These data were presented in Fig. 3E.

**Table S5. The mutation rate of individual nucleotides detected by SHAPE assay using 1M7 as probe.**

These data were presented in Fig. 6F-G, and S10A.

**Table S6. The mutation rate of individual nucleotides detected by SHAPE assay using 5NIA as probe.**

These data were presented in Fig. S10B-D.
