# Supplementary material for: The lupus autoantigen La/Ssb is an Xist-binding protein involved in Xist folding and cloud formation
Source: Nucleic Acids Res. 2021 Nov 1;49(20):11596–613. doi: 10.1093/nar/gkab1003 (PMC8599922; doi:10.1093/nar/gkab1003)
Supplement: gkab1003_Supplemental_Files [file gkab1003_supplemental_files.zip › Supplementary Materials final submission.pdf]

Supplementary Information

The lupus autoantigen La/Ssb is an *Xist*-binding protein involved in *Xist* folding and cloud formation

Norbert Ha[2,4], Nan Ding[1,4], Ru Hong[2], Rubing Liu[2], Xavier Roca[2], Yingyuan Luo[1], Xiaowei Duan[1], Xiao Wang[1], Peiling Ni[1], Haiyang Wu[3], Li-Feng Zhang[2,3,*] & Lingyi Chen[1,*]

[1]Institute of Translational Medicine, Tianjin Union Medical Center, Collaborative Innovation Center for Biotherapy, Collaborative Innovation Center of Tianjin for Medical Epigenetics, Tianjin Key Laboratory of Protein Sciences, National Demonstration Center for Experimental Biology Education and College of Life Sciences, Nankai University, Tianjin 300071, China. [2]School of Biological Sciences, Nanyang Technological University, 60 Nanyang Drive, Singapore 637551. [3]TCRCure Biological Technology Co Ltd., Guangdong, China. [4]These authors contributed equally to this work.

[*]**Correspondence:** Lingyi Chen, lingyichen@nankai.edu.cn

Li-Feng Zhang, ZLF02472@gmail.com

Keywords: La, *Ssb*, lncRNA in-cell structure, *Xist*, X chromosome inactivation.

Author Contributions: N.H., N.D., R.H., R.L., Y.L., X.D., X.W., and P.N. performed experiments, N.H., X.R., H.W., L-F.Z. and L.C. analyzed the data, L-F.Z. and L.C. designed the experiments and wrote the paper.

This PDF file includes:

Supplementary text
Figures S1 to S10
Table S1
SI References

Other supplementary materials for this manuscript include the following:

Tables S2 to S6
Movies S1 to S3

Supplementary Information text

Comparison of the candidate proteins of this study with previous studies:

All 81 proteins identified by Chu et al (1) (annotated as CC in Table S1) and all 10 proteins identified by McHugh et al (2) (annotated as MC in Table S1) were compared with the candidate proteins identified in this study. The study of Minajigi et al (3) (annotated as MA in Table S1) identified more than 700 proteins. The top 93 proteins ranked by “log2_avgFX/avgMX” were selected and compared with the candidate proteins identified in this study.

Definition of functional groups:

The candidate proteins are classified into each functional group based on the protein’s function description in Uniport and NCBI gene.

<u>Nuclear actin and related proteins:</u>

1) The protein is myosin, unconventional myosin or myosin-binding.
2) The protein is actin-like, actin-related, actin-binding, actin filament binding, actin-crosslinking or regulates actin dynamics and organization.

<u>Chromatin and related proteins:</u>

1) The protein is involved in regulating chromatin structure, recruiting chromatin-remodeling enzymes, mediating nuclear import of histones and nucleosome assembly.
2) The protein possesses, recruits or regulates histone modification enzyme activities.
3) The protein binds to histones or modified histones.
4) The protein is a histone or histone variant.

<u>DNA and RNA binding:</u>

1) The protein is RNA-binding or DNA-binding.

<u>Nuclear RNPs, ribosomal and nucleolar proteins:</u>

1) The protein is a nuclear RNP.
2) The protein is a ribosomal protein.
3) The protein is a nucleolar protein.
4) The protein is involved in processing rRNA.

<u>Membrane Proteins:</u>

1) The protein is associated with plasma membrane, mitochondria membrane, trans-Golgi network and vesicle trafficking

Supplemental Figures

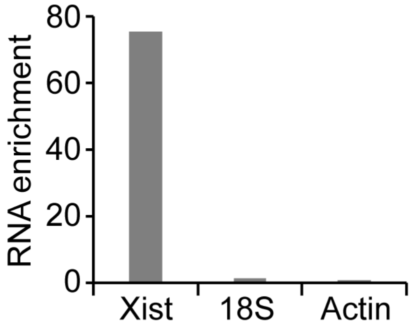

Figure S1. Enrichment of *Xist* RNA after FLAG IP. FLAG IP was performed as described in Figure 1B. RNA isolated from the FLAG IP sample before benzonase treatment, was analysed by qRT-PCR, normalized to input RNA sample. The enrichment of *Xist*, *18S*, and *Actin* RNA were presented in the plot.

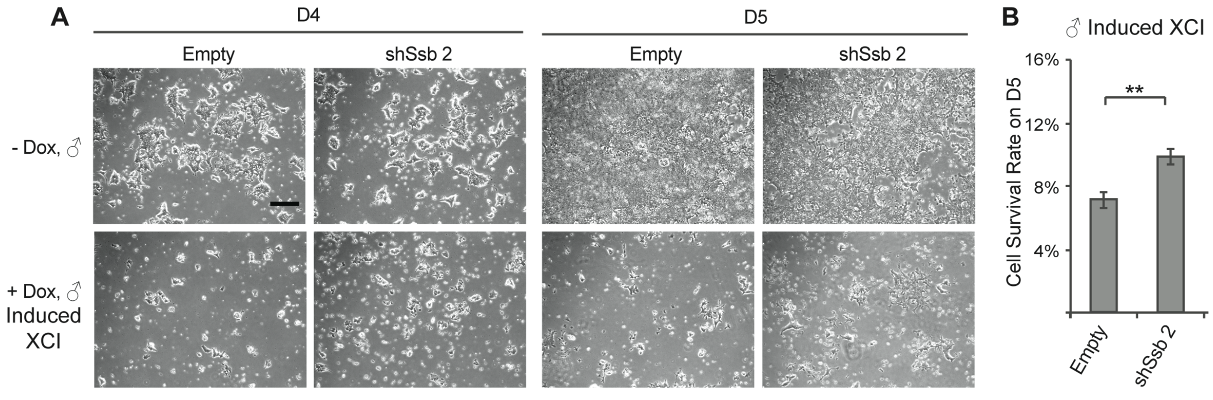

Figure S2. The effects of *Ssb* knockdown on the induced XCI in differentiating male ES cells. **(A)** Representative bright field microscope images of differentiating ES cell treated with Doxycycline for 4 and 5 days (D4 & D5). Clonal ES cell lines stably transfected with the shRNA constructs against *Ssb* and the empty shRNA vector are shown. Doxcycycline treatment was started upon *in vitro* differentiation. Scale bars, 300 μm. **(B)** Cell survival rate was calculated by cell counting on day 5. Data are shown as mean ± SEM. The statistical analysis used is the Student's *t*-test. **p < 0.01; n = 3.

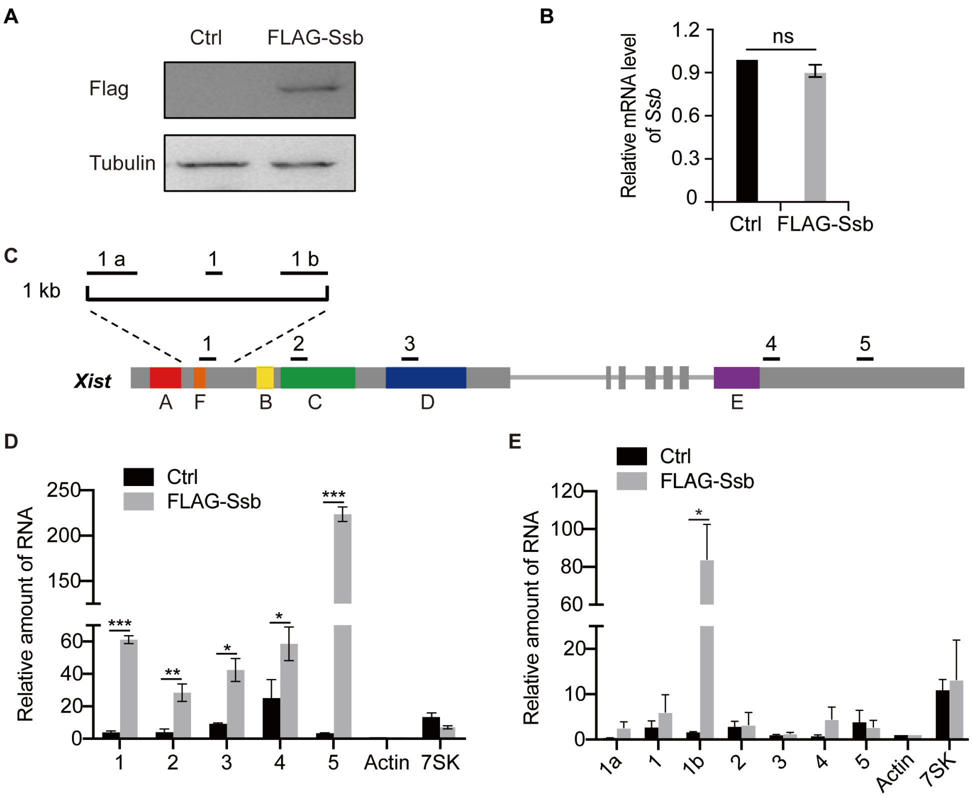

Figure S3. RIP and CLIP assays to validate the interaction between Ssb and *Xist* RNA. (A) Western blot showing the ectopic expression of FLAG-Ssb. (B) Overall expression levels of *Ssb* mRNA are similar in *Xist* inducible male ES cell lines with or without ectopic expression of FLAG-Ssb, analysed by quantitative RT-PCR. (C) A schematic illustration of the *Xist* locus. Repeat A-F are shown in coloured boxes. Amplicons of the PCR reactions are marked with short bars 1-5. A 1-kb region around amplicon 1 is shown in the blowup to illustrate neighbouring amplicons 1a and 1b. (D) and (E) *Xist* inducible male ES cell lines with or without ectopic expression of FLAG-Ssb were treated with doxycycline treatment for 24 hours, and subjected to RIP (D) and CLIP (E) experiments using anti-FLAG M2 beads. RIP and CLIP RNA were quantified by qRT-PCR. Normalization was performed using *Actb*. Error bars indicate SEM (n = 3). The statistical analysis used is the Student's *t*-test. *p < 0.05; **p < 0.01; ***p < 0.001.

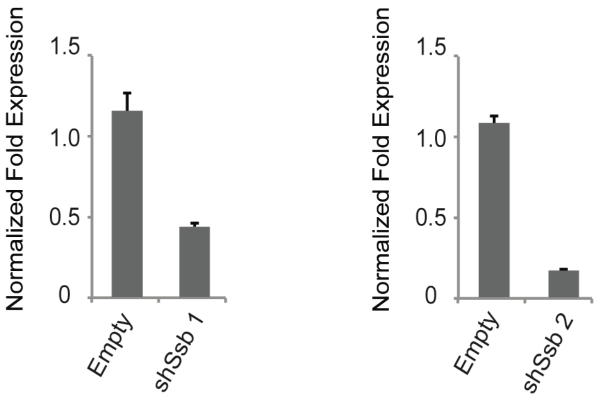

Figure S4. Quantitative RT-PCR to assess the effect of shRNA knockdown of *Ssb* in the female 3F1 cell lines. Data are shown in relative fold expression. Normalization was performed using *Gapdh*. Error bars indicate SEM (n = 3).

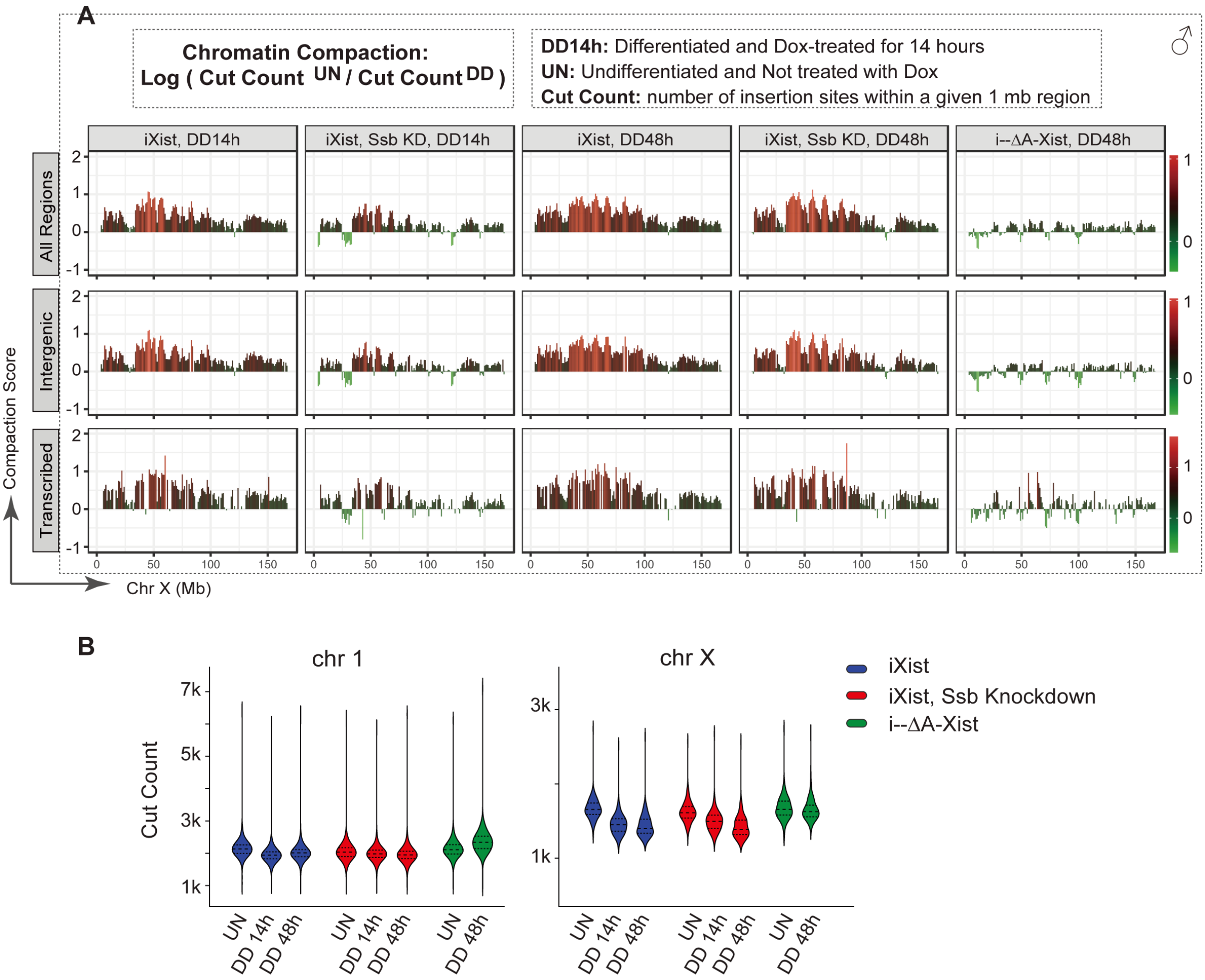

Figure S5. ATAC-seq results. (A) ATAC-seq results as described in Figure 3D. The chromatin compaction scores of each 1Mb region are shown as bars colored by value. For detailed analysis, the genomic regions are further categorized as intergenic and transcribed regions. i-ΔA-*Xist* is an inducible mutant *Xist* transgene, in which the critical A-repeat region is deleted. **(B)** The chromosomes are divided into 1-mb regions and the Cut Counts of each 1-Mb region are shown in violin plots. The analysis was done using 10 million reads randomly selected from each sample (SRA accession number PRJNA545157).

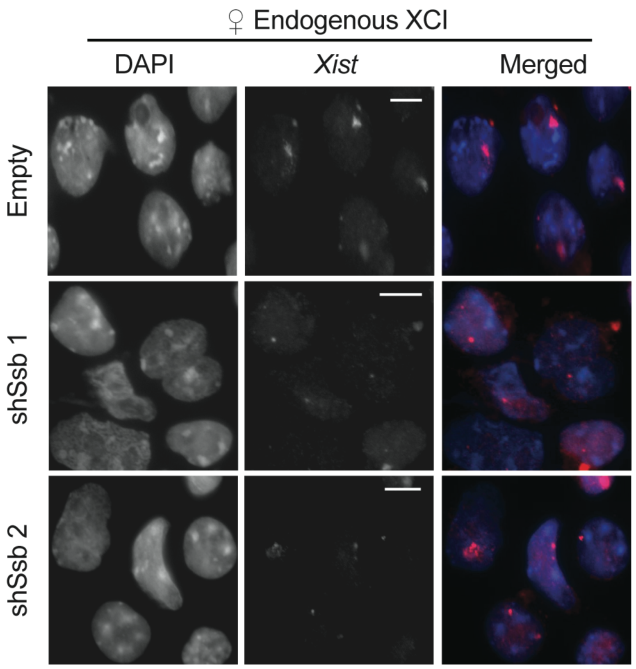

Figure S6. *Xist* RNA FISH results. The experiment was performed using day 6 *in vitro* differentiating female ES cells. DNA was counter stained with DAPI (blue). Scale bars, 8 μm.

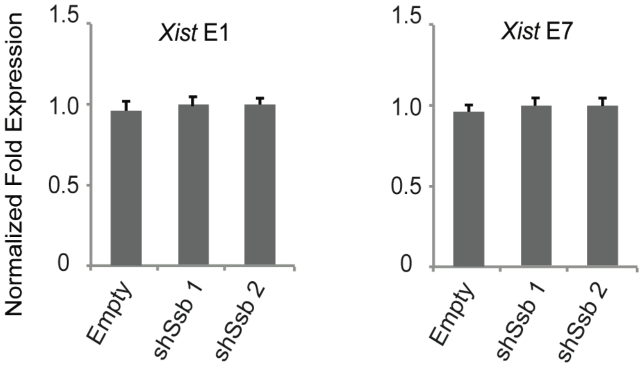

Figure S7. Quantitative RT-PCR to assess the expressions of *Xist* RNA in the *Xist* inducible male ES cell line upon *Ssb* knock-down. Cells were cultured as ES cells and Doxycycline treatment was carried out for 14 hours. Primers used were either targeting *Xist* Exon 1 (E1) or Exon 7 (E7). Data are shown in relative fold expression. Normalization was performed using *Gapdh*. Error bars indicate SEM (n = 6 for Empty; n = 3 for shSsb 1 and ShSsb 2).

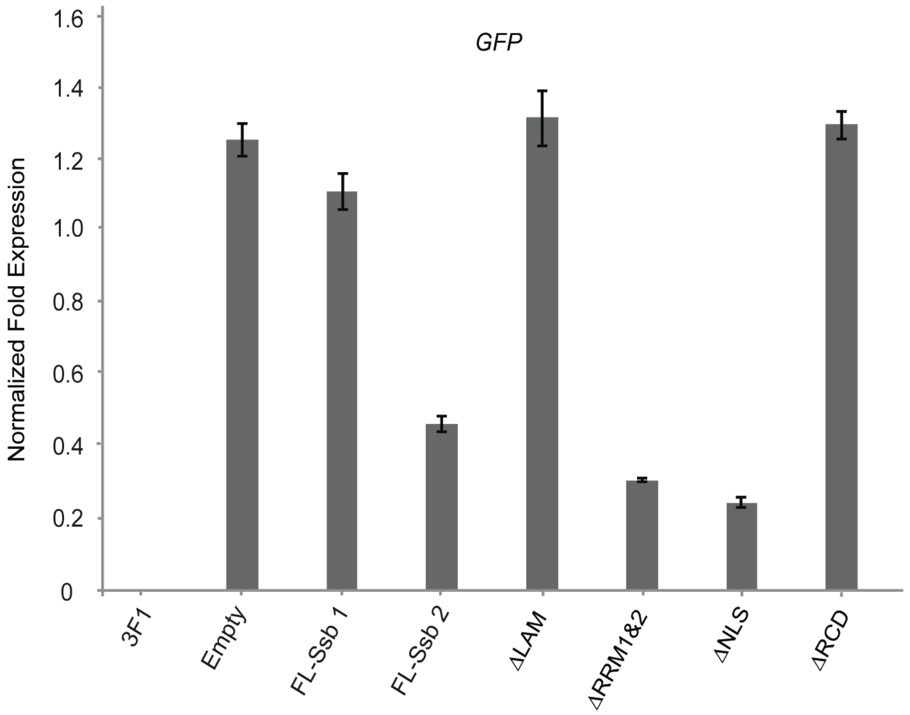

Figure S8. Quantitative RT-PCR to assess the expression level of the GFP-fusion genes in the stable rescue lines. Data are shown in relative fold expression. Normalization was performed using *Gapdh*. Error bars indicate SEM (n = 3).

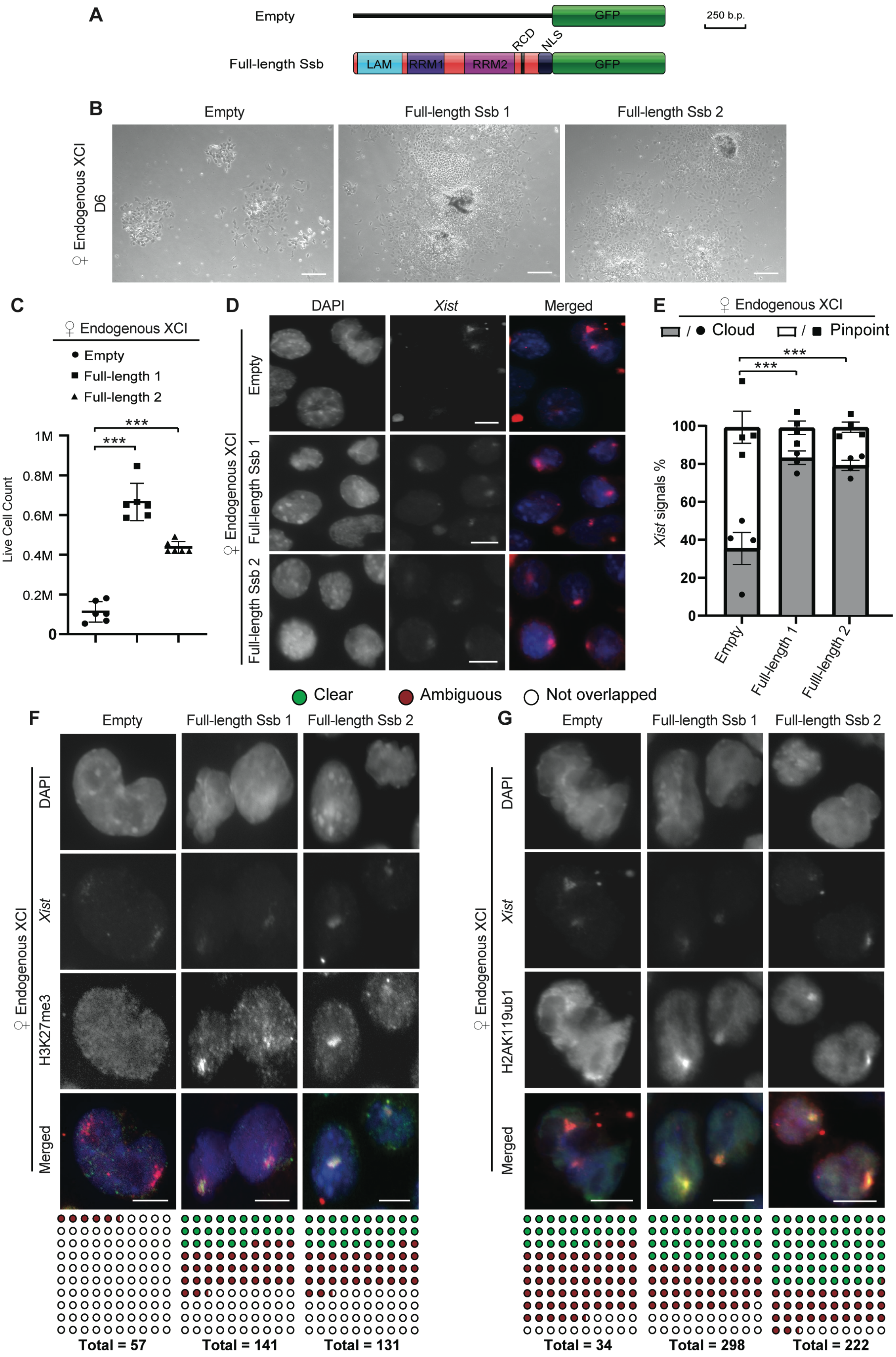

Figure S9. The rescue effects of full-length *Ssb* constructs on *Ssb* knockdown female cells during *in vitro* differentiation. (A) Diagram of the functional domains of Ssb and the plasmid constructs. **(B)** Representative microscope images of day 6 differentiating ES cells. Scale bars, 300

μm. Cells are clonal ES cell lines stably transfected with the corresponding plasmid constructs. **(C)** The rescue effects of full-length *Ssb* constructs on cell survival of *Ssb* knockdown cells during *in vitro* differentiation. Cell counts of day 6 *in vitro* differentiation are shown. Data are shown as mean ± SEM (n = 6). The statistical analysis used is the Student's *t*-test. The data pairs with $p > 0.05$ (n.s.) are labeled. ***$p < 0.001$. **(D)** *Xist* RNA FISH. DNA was counterstained with DAPI. Scale bars, 8μm. **(E)** Quantification of *Xist* RNA FISH signals. Data are shown as mean ± SEM (n = 243 for Empty; n = 545 for Full-length 1; n = 839 for Full-length 2). ***$p < 0.001$ by X^2 test. **(F and G)** Immuno-RNA FISH detecting H2AK119ub, H3K27me3 and *Xist.* Cells were *in vitro* differentiated for 6 days. Immunostains were performed before the RNA FISH. DNA was counter stained with DAPI (blue). Scale bars, 8 μm. The total number of *Xist* clouds with clear, ambiguous or undetectable overlapping with the histone mark enrichment were tallied and tabulated below.

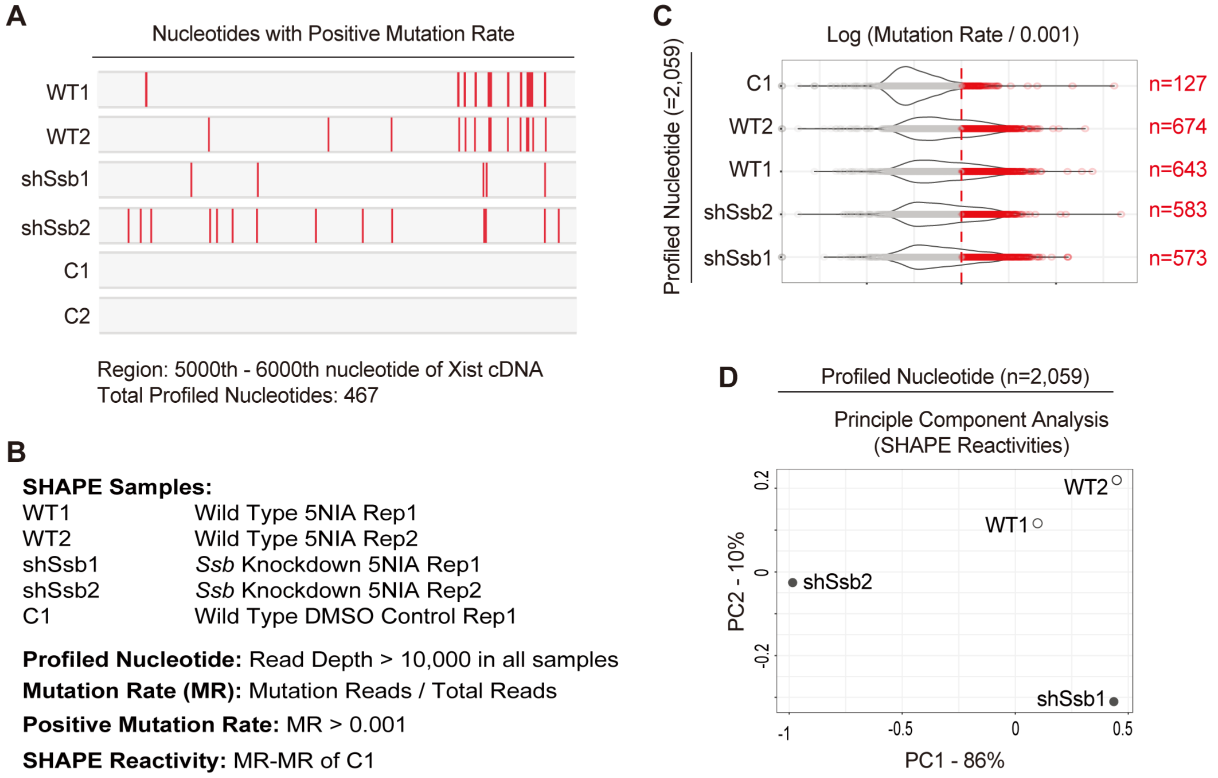

Figure S10. Altered *Xist* RNA folding in *Ssb* knockdown cells revealed by SHAPE experiments. (A) The distribution pattern of the profiled nucleotides by 1M7 with positive mutation rates along the region of 5000th to 6000th nucleotide of mouse *Xist* cDNA. Only profiled nucleotides with positive mutation rates are shown. The nucleotides are arranged in a sequential order based on nucleotide positions along *Xist*. Each nucleotide is represented by a red bar. **(B)** Sample identities of SHAPE experiments using 5NIA as probe, and parameters used in data analysis. **(C)** Mutation rates of the 2,059 profiled nucleotides of each sample. **(D)** A PCA plot of the SHAPE reactivity profiles using 5NIA as probe.

Table S1 *Xist*-binding proteins identified by "FLAG-out"

Nuclear Actin and Related Proteins		
ID	Name	Also Identified in
Q8VDD5	Myosin-9	
Q8BFZ3	Beta-actin-like protein 2	
Q9QXZ0	Microtubule-actin cross-linking factor 1	
Q80X90	Filamin-B	
Q9JI91	Alpha-actinin-2	
Q6URW6	Myosin-14	
Q9JMH9	Unconventional myosin-XVIIIa	
Q9JKF1	Ras GTPase-activating-like protein IQGAP1	
Q91Z67	SLIT-ROBO Rho GTPase-activating protein 2	
Q5NBX1	Protein cordon-bleu	
E9Q634	Unconventional myosin-Ie	
P59242	Cingulin	
P46735	Unconventional myosin-Ib	
Q8BQ30	Phostensin	
Q6R891	Neurabin-2	
Q8CI43	Myosin light chain 6B	
Q9Z2N8	Actin-like protein 6A	
Q9QXS6	Drebrin	
Q9R0P5	Destrin	
Q6P9R2	Serine/threonine-protein kinase OSR1	
Q61553	Fascin	
Q9WTI7	Unconventional myosin-Ic	
Q61879	Myosin-10	
Q8BTM8	Filamin-A	
Q64331	Unconventional myosin-VI	
Q9DBR7	Protein phosphatase 1 regulatory subunit 12A	
Q9ERG0	LIM domain and actin-binding protein 1	
Q9QYC0	Alpha-adducin	
Q9CVB6	Actin-related protein 2/3 complex subunit 2	
Q99JY9	Actin-related protein 3	
P14602	Heat shock protein beta-1	
Q2KN98	Cytospin-A	
P58774	Tropomyosin beta chain	
Q9QYB5	Gamma-adducin	
Q3THE2	Myosin regulatory light chain 12B	
O88990	Alpha-actinin-3	
O70318	Band 4.1-like protein 2	
Q9JJ28	Protein flightless-1 homolog	

Chromatin and Related Proteins		
ID	Name	Also Identified in
P62141	Serine/threonine-protein phosphatase PP1-beta catalytic subunit	
Q7TPV4	Myb-binding protein 1A	CC, MA
Q78ZA7	Nucleosome assembly protein 1-like 4	
O35129	Prohibitin-2	
O09106	Histone deacetylase 1	

Q99LL5	Periodic tryptophan protein 1 homolog	
P10853	Histone H2B type 1-F/J/L	
Q99J09	Methylosome protein 50	
Q60972	Histone-binding protein RBBP4	
Q62318	Transcription intermediary factor 1-beta	
P02301	Histone H3.3C	
Q9WTM5	RuvB-like 2	
P70168	Importin subunit beta-1	

**DNA and RNA Binding Proteins**		
**ID**	**Name**	**Also Identified in**
P17225	Polypyrimidine tract-binding protein 1	CC, MC
Q60817	Nascent polypeptide-associated complex subunit alpha	
Q8R4U7	Leucine zipper protein 1	
Q80U78	Pumilio homolog 1	
P11031	Activated RNA polymerase II transcriptional coactivator p15	
Q9CY58	Plasminogen activator inhibitor 1 RNA-binding protein	
Q921F2	TAR DNA-binding protein 43	CC, MA
P53996	Cellular nucleic acid-binding protein	
Q921M3	Splicing factor 3B subunit 3	
Q9DBD5	Proline-, glutamic acid- and leucine-rich protein 1	
P26369	Splicing factor U2AF 65 kDa subunit	
Q8K3Y3	Protein lin-28 homolog A	CC
Q9CQF3	Cleavage and polyadenylation specificity factor subunit 5	
Q3U1J4	DNA damage-binding protein 1	
Q99KP6	Pre-mRNA-processing factor 19	
Q60865	Caprin-1	
Q02248	Catenin beta-1	
Q8BG81	Polymerase delta-interacting protein 3	CC
P97855	Ras GTPase-activating protein-binding protein 1	
P32067	Ssb (Lupus La protein homolog)	CC

**Membrane Proteins**		
**ID**	**Name**	**Also Identified in**
P39447	Tight junction protein ZO-1	
Q9Z0U1	Tight junction protein ZO-2	
P48962	ADP/ATP translocase 1	
Q68FD5	Clathrin heavy chain 1	
P23242	Gap junction alpha-1 protein	
Q9WVE8	Protein kinase C and casein kinase substrate in neurons protein 2	
P17809	Solute carrier family 2, facilitated glucose transporter member 1	
Q7TMK6	Protein Hook homolog 2	

**Nuclear RNP; Ribosomal or Nucleolar Proteins**		
**ID**	**Name**	**Also Identified in**
P47962	60S ribosomal protein L5	
P35980	60S ribosomal protein L18	

P62911	60S ribosomal protein L32	
P62267	40S ribosomal protein S23	
O09167	60S ribosomal protein L21	
P62862	40S ribosomal protein S30	
P62717	60S ribosomal protein L18a	
P12970	60S ribosomal protein L7a	
P41105	60S ribosomal protein L28	
P47911	60S ribosomal protein L6	
P62855	40S ribosomal protein S26	
P61514	60S ribosomal protein L37a	
P47915	60S ribosomal protein L29	
P14148	60S ribosomal protein L7	
P53026	60S ribosomal protein L10a	
Q9D8E6	60S ribosomal protein L4	
P62242	40S ribosomal protein S8	
P14206	40S ribosomal protein SA	
P47963	60S ribosomal protein L13	
P63276	40S ribosomal protein S17	
P47964	60S ribosomal protein L36	
P62892	60S ribosomal protein L39	
Q9D0E1	Heterogeneous nuclear ribonucleoprotein M	CC, MC, MA
Q9Z2X1	Heterogeneous nuclear ribonucleoprotein F	
Q60668	Heterogeneous nuclear ribonucleoprotein D0	CC
Q8VEK3	Heterogeneous nuclear ribonucleoprotein U	CC, MC, MA
Q61937	Nucleophosmin	
Q9CPP0	Nucleoplasmin-3	
O54825	Bystin	
Q62189	U1 small nuclear ribonucleoprotein A	

Other Proteins		
**ID**	**Name**	**Also Identified in**
O70251	Elongation factor 1-beta	
O54931	A-kinase anchor protein 2	
Q9D8W5	26S proteasome non-ATPase regulatory subunit 12	
O88844	Isocitrate dehydrogenase [NADP] cytoplasmic	
O88735	Ensconsin	
Q9R0Q9	Mannose-P-dolichol utilization defect 1 protein	
Q61316	Heat shock 70 kDa protein 4	
Q9JKV1	Proteasomal ubiquitin receptor ADRM1	
Q8BFR5	Elongation factor Tu, mitochondrial	
Q9CZS1	Aldehyde dehydrogenase X, mitochondrial	
Q8BJM7	S-adenosyl-L-methionine-dependent tRNA 4-demethylwyosine synthase	
P45376	Aldose reductase	
P23116	Eukaryotic translation initiation factor 3 subunit A	
P26516	26S proteasome non-ATPase regulatory subunit 7	
P17751	Triosephosphate isomerase	
O88685	26S protease regulatory subunit 6A	
P05064	Fructose-bisphosphate aldolase A	
P68040	Guanine nucleotide-binding protein subunit beta-2-like 1	

Q9D4J1	EF-hand domain-containing protein D1	
Q8QZY1	Eukaryotic translation initiation factor 3 subunit L	
Q8JZQ9	Eukaryotic translation initiation factor 3 subunit B	
P60229	Eukaryotic translation initiation factor 3 subunit E	
P35700	Peroxiredoxin-1	
P17182	Alpha-enolase	
Q91VJ4	Serine/threonine-protein kinase 38	
P24369	Peptidyl-prolyl cis-trans isomerase B	
Q3TXS7	26S proteasome non-ATPase regulatory subunit 1	
P99024	Tubulin beta-5 chain	
P01868	Ig gamma-1 chain C region secreted form	

CC: (1), MC: (2), MA: (3).

Movies S1: The emergence of induced *Xist* RNA signals in wild type differentiating ES cells.
Movies S2, S3: The emergence of induced *Xist* RNA signals in *Ssb* mutant differentiating ES cells.

Table S2. *Xist* binding proteins identified by FLAG-out and mass spectrometry.
(A) Proteins identified in the i-Empty sample. (B) Proteins identified in the i-FLAG-*Xist* sample. (C) Proteins identified in i-FLAG-*Xist*, but not in i-Empty. (D) Proteins identified in both i-Empty and i-FLAG-*Xist*.

Table S3. The cut count and compaction score of ATAC-seq on chromosome 1 and X.
Cut counts (A and C) and compaction scores (B and D) were calculated for regions on chromosome 1 (A and B) and Chromosme X (C and D). These data were presented in Fig. 3D and Fig. S5.

Table S4. The allelotype score for individual SNPs detected by padlock SNP capture.
These data were presented in Fig. 3E.

Table S5. The mutation rate of individual nucleotides detected by SHAPE assay using 1M7 as probe.
These data were presented in Fig. 6F-G, and S10A.

Table S6. The mutation rate of individual nucleotides detected by SHAPE assay using 5NIA as probe.
These data were presented in Fig. S10B-D.

Supplementary References

1. Chu, C., Zhang, Q.C., da Rocha, S.T., Flynn, R.A., Bharadwaj, M., Calabrese, J.M., Magnuson, T., Heard, E. and Chang, H.Y. (2015) Systematic discovery of Xist RNA binding proteins. *Cell*, **161**, 404-416.
2. McHugh, C.A., Chen, C.K., Chow, A., Surka, C.F., Tran, C., McDonel, P., Pandya-Jones, A., Blanco, M., Burghard, C., Moradian, A. *et al.* (2015) The Xist lncRNA interacts directly with SHARP to silence transcription through HDAC3. *Nature*, **521**, 232-236.
3. Minajigi, A., Froberg, J.E., Wei, C., Sunwoo, H., Kesner, B., Colognori, D., Lessing, D., Payer, B., Boukhali, M., Haas, W. *et al.* (2015) Chromosomes. A comprehensive Xist interactome reveals cohesin repulsion and an RNA-directed chromosome conformation. *Science*, **349**, aab2276.
